# Investigating the risks of removing wild meat from global food systems

**DOI:** 10.1016/j.cub.2021.01.079

**Published:** 2021-04-26

**Authors:** Hollie Booth, Michael Clark, E.J. Milner-Gulland, Kofi Amponsah-Mensah, André Pinassi Antunes, Stephanie Brittain, Luciana C. Castilho, João Vitor Campos-Silva, Pedro de Araujo Lima Constantino, Yuhan Li, Lessah Mandoloma, Lotanna Micah Nneji, Donald Midoko Iponga, Boyson Moyo, James McNamara, O. Sarobidy Rakotonarivo, Jianbin Shi, Cédric Thibaut Kamogne Tagne, Julia van Velden, David R. Williams

**Affiliations:** 1 The Interdisciplinary Centre for Conservation Science (ICCS), Department of Zoology, University of Oxford, Oxford, UK; 2 Oxford Martin School and Nuffield Department of Population Health, University of Oxford, Oxford, UK; 3 Centre for African Wetlands, University of Ghana, Legon, Accra, Ghana; 4 Department of Ecology, National Institute of Amazonian Research, Brazil; 5 RedeFauna—Rede de Pesquisa em Diversidade, Conservação e Uso da Fauna da Amazônia, 70879-070, Brasília, DF, Brazil; 6 Ethnoconservation and Protected Areas Laboratory, State University of Santa Cruz, Ilhéus, Bahia, Brazil; 7 Faculty of Ecology and Natural Resource Management, Norwegian University of Life Sciences, 1430 Ås, Norway; 8 Instituto de Ciências Biológicas e da Saúde, Universidade Federal de Alagoas, AL Maceió, Brazil; 9 Lilongwe University of Agriculture and Natural Resource, Malawi; 10 Kunming Institute of Zoology, Chinese Academy of Sciences, PR China; 11 Institut de recherche en ecologie tropicale, Centre National de la Recherche Scientifique et Technologique, Libreville, Gabon; 12 Conservation Research Consultants, London, UK; 13 Ecole Supérieure des Sciences Agronomiques, Université d’Antananarivo, Madagascar; 14 School of Environment, Beijing Normal University Beijing 100875, China; 15 Fondation Camerounaise Terre Vivante, Cameroon; 16 Environmental Futures research institute, Griffith University, Australia; 17 Sustainability Research Institute, School of Earth and Environment, University of Leeds, Leeds, UK

**Keywords:** wild meat, land use, biodiversity conservation, food security, public health, bush meat, wildlife trade, policy, food systems, infectious dieases

## Abstract

The COVID-19 pandemic has brought humanity’s strained relationship with nature into sharp focus, with calls for cessation of wild meat trade and consumption, to protect public health and biodiversity.^1^^,^^2^ However, the importance of wild meat for human nutrition, and its tele-couplings to other food production systems, mean that the complete removal of wild meat from diets and markets would represent a shock to global food systems.^3^, ^4^, ^5^, ^6^ The negative consequences of this shock deserve consideration in policy responses to COVID-19. We demonstrate that the sudden policy-induced loss of wild meat from food systems could have negative consequences for people and nature. Loss of wild meat from diets could lead to food insecurity, due to reduced protein and nutrition, and/or drive land-use change to replace lost nutrients with animal agriculture, which could increase biodiversity loss and emerging infectious disease risk. We estimate the magnitude of these consequences for 83 countries, and qualitatively explore how prohibitions might play out in 10 case study places. Results indicate that risks are greatest for food-insecure developing nations, where feasible, sustainable, and socially desirable wild meat alternatives are limited. Some developed nations would also face shocks, and while high-capacity food systems could more easily adapt, certain places and people would be disproportionately impacted. We urge decision-makers to consider potential unintended consequences of policy-induced shocks amidst COVID-19; and take holistic approach to wildlife trade interventions, which acknowledge the interconnectivity of global food systems and nature, and include safeguards for vulnerable people.

## Highlights

• 15 countries identified as at risk of food insecurity from wild meat prohibitions
• Extra agricultural land to replace wild meat protein with livestock is ∼124,000 km^2^
• This land-use change could drive >260 species toward extinction, globally
• Context-specific factors moderate risks of food insecurity and biodiversity loss

## Graphical abstract

**Figure:**
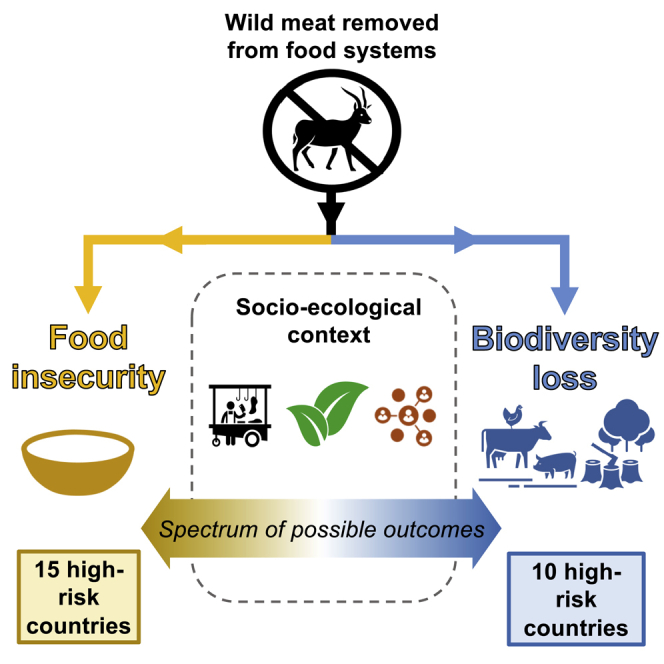

## Teaser

The COVID-19 pandemic has led to calls to prohibit wild meat consumption, to protect public health and biodiversity. However, Booth et al. demonstrate that the sudden removal of wild meat from food systems could negatively impact people and nature. Wildlife trade policy interventions need to consider tele-couplings between food systems and nature.

## Results

### A global perspective on the potential negative consequences of removing wild meat from food systems

To investigate the potential negative consequences of the sudden policy-induced loss of wild meat from food systems (e.g., due to prohibitions on wild meat trade and consumption in response to COVID-19), we explored global patterns in two contrasting ‘worst-case scenarios’. A worst-case scenario for food insecurity is one in which all wild meat is suddenly removed from food systems, in the absence of feasible, socially desirable alternatives, such that the lost protein and nutrients are not replaced. Conversely, if all wild meat is replaced by animal agriculture, this could lead to a worst-case land-use change scenario, with subsequent impacts on biodiversity loss and the risk of emerging infectious diseases (EIDs). High-quality data on wild meat consumption at a global scale is limited. However, by drawing together available global datasets on nutrient supply and land demand for biodiversity^4^^,^^7^, ^8^, ^9^, ^10^, ^11^ we provide a rudimentary estimate of the animal protein that would be lost from diets if all wild meat consumption ceased, and the land required to replace this protein with livestock production, for 83 countries.

### Food insecurity

**Figure 1:**
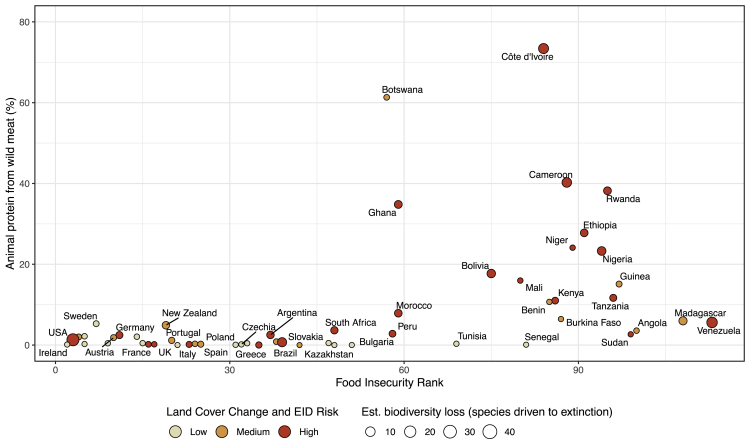
Summarizing global patterns in the risk of negative consequences of bans on wildlife trade and consumption for 54 countries Countries at high risk of food insecurity are located in the top right-hand corner (e.g., Côte D’Ivoire and Botswana) and extreme right of the figure (e.g., Madagascar, where per capita protein intake could fall below minimum healthy intake, as recommended by the World Health Organization; as per Figure S1). Countries at highest risk of land use change, biodiversity loss and elevated EID risk are larger red circles. Countries which are both in the top right hand-corner and have larger red circles could face the severest trade-offs between lost protein, or land-use change and a loss of biodiversity to replace the protein. See Tables S1 and S2 for data, and STAR methods for data sources. N.B. Several countries known to have high wild meat consumption (e.g., Sierra Leone, Gabon, DR Congo, Uganda) are not included here due to lack of data, while no food insecurity rank was available for Republic of Congo, Zimbabwe and Central African Republic.

The sudden loss of wild meat from national food systems, and the ability of countries’ food systems to absorb these shocks, are unequally distributed, with risks of protein shortfalls in some of the world’s most food-insecure countries. We identified 15 countries at high risk of food insecurity, which rely on wild meat for more than 5% of total animal protein, and are currently ranked in the bottom 50% of the global food security index (Figure 1; Table S1). Overall, Côte d’Ivoire and Botswana were identified as having the highest reliance on wild meat, deriving 73% and 61% of animal protein from wild meat, respectively, and ranking 84^th^ and 57^th^ (out of 113) for global food insecurity, respectively. Eight countries could be at especially high risk of protein deficiencies, because loss of wild meat without immediate replacement could cause mean per capita protein supplies to fall below World Health Organization (WHO) recommended minimum intakes. These countries, all of which are in Sub-Saharan Africa, are: Madagascar, Republic of Congo, Guinea, Rwanda, Central African Republic, Zimbabwe, Botswana, Côte d’Ivoire (Figure S1). Prohibitions on wildlife use could exacerbate existing food insecurity in these countries, especially if implemented without rapid provision of alternatives. However, wild meat consumption is not limited to food-insecure countries: 10 countries which are members of the Organization for Economic Co-operation and Development (OECD), and therefore have high-income economies/very high Human Development Indexes, source at least 1% of protein from wild meat. These countries are: Austria, Colombia, Denmark, Germany, New Zealand, Norway, Portugal, Switzerland, Sweden, and the USA, with the USA being the world’s third largest reported wild meat consumer in absolute terms (53.6 million kg per year), only superseded by Nigeria and Côte d’Ivoire (62.2 and 58.8 million kg per year, respectively) (Figure 1; Table S1). However, low levels of food insecurity/higher food system resilience suggest these countries’ food systems could more easily adapt to loss of wild meat (Figure 1).

### Land-use change and biodiversity loss

We estimated that 123,980 km^2^ of additional agricultural land would be needed to replace wild meat protein with protein from domestic livestock (based on region- and livestock-specific estimates of land demand per unit livestock production, and current livestock consumption) (Table S2). We identified two countries where estimated demand for new agricultural land was over 10,000 km^2^: Nigeria (10,320 km^2^) and USA (12,282 km^2^); and a further seven with 5 - 10,000 km^2^: Brazil, Colombia, Ethiopia, Ecuador, Côte d’Ivoire, Bolivia, and Venezuela (Table S2).

Based on country-specific estimates of the species extinction risks associated with this land use change (i.e., the number of species destined to be set on a track toward extinction from agricultural land and land-use change), we estimate that up to 267 species could be driven toward extinction globally, with wide variation in potential biodiversity impacts across countries (Table S2). For many countries, extinction estimates are low (i.e., less than one extinction), however, in the top 10 extinction-estimate countries, at least five species are destined for extinction, with some as high as 40-80 species per country. These top 10 countries are primarily located in South America (Ecuador [85.1 species destined for extinction], Colombia [41.8 species], Venezuela [15.1 species], Brazil [8.2], Bolivia [5.9], and Suriname [6.2]) and Sub-Saharan Africa (Côte d’Ivoire [12.4 species destined toward extinction], Cameroon [10 species], and Nigeria [6.3 species]) as well as the USA (with the third highest number of estimated extinctions, globally: 24.8 species).

Area and rate of increase of pasture and cropland, and absolute livestock and poultry numbers, are also significant predictors of emerging infectious disease (EID) occurrence.^10^ As such, rapid increases in land area for animal agriculture may bring elevated EID risk (Figure 1). These risks are further exacerbated in forest regions with high mammalian biodiversity—a classification which includes many of the countries with the highest estimated land demands of replacing wild caught meat.^10^^,^^12^

### Case studies

In reality, the impacts of prohibitions on wild meat consumption would be moderated by context-specific factors. Acknowledging this, we qualitatively analyzed 10 case studies across a range of contexts, to explore likely outcomes in different places, under different ecological and socio-economic conditions. The cases that may find it most difficult to adapt are represented by Madagascar, rural Gabon, the East Region of Cameroon, Malawi, and the Brazilian Amazon. In these places, wild meat consumption forms an important component of people’s diets, and substitutes are not readily available for a range of environmental and socio-economic reasons^13^, ^14^, ^15^, ^16^, ^17^, ^18^ (Table 1, Table S3). However, the lack of viable alternatives, combined with epistemic dissonance, social illegitimacy due to food security trade-offs, and limited enforcement capacity suggest that non-compliance with prohibitions is also likely, such that wild meat consumption may continue illicitly^19^ (Table 1, Table S3). Efforts to reduce wild meat consumption will likely require the identification and gradual introduction of alternative protein and nutrient sources in these areas, using participatory approaches to ensure their legitimacy and uptake.^14^

**Table 1:** Summary of descriptive case studies for 10 places

Case study	Current consumption/dependence on wild meat	Resilience and adaptability		Overall outlook	Key refs
		Eco-logical	Socio-economic		
**Madagascar**	Ubiquitous and very high	Very Low	Very Low	Food system would struggle to adapt; protein intake may fall leading to malnutrition. Prohibitions may be socially illegitimate and difficult to enforce.	^18^^,^^20^
**East Region, Cameroon**	Ubiquitous and high	Low	Low	Rural food system would struggle to adapt. Prohibitions may be socially illegitimate and difficult to enforce.	^16^^,^^21^
**Malawi**	Moderate, dependence varies in urban versus rural	Low	Low	Rural food system would struggle to adapt, additional prohibitions may be socially illegitimate, with persistence of informal markets. Urban Malawians consuming wild meat (mice and birds) as delicacies may adapt.	^17^^,^^22^
**Rural Gabon**	Ubiquitous and high	Low	Very Low	Rural food system would struggle to adapt. Urbanisation reduces hunting, though demand may remain due to increased wealth and preferences. Prohibitions may be socially illegitimate and difficult to enforce, even with alternatives.	23, 24, 25
**Brazilian Amazon**	Ubiquitous and high	High	Very Low	Rural and indigenous food system would struggle to adapt. Reliance on fishing may increase, agricultural expansion may occur to supply urban consumers. High social costs for rural and indigenous peoples, prohibitions difficult to enforce.	^13^^,^^26^
**Brazilian Atlantic Forest**	Moderate	Moderate	Moderate	Food system could potentially adapt; though agricultural expansion should focus on intensification of production and recovery of degraded areas to avoid further deforestation and threats to biodiversity. Social costs would be high for rural poor and indigenous populations. Current prohibitions are already difficult to enforce.	27, 28, 29
**Tropical SW Ghana**	Moderate	Moderate	Moderate	Food system could potentially adapt overall; however severe impacts would be felt by some. Economic shocks may be the biggest risk, for female traders/wholesalers.	^24^^,^^30^^,^^31^
**USA**	Low overall, relatively high in some areas	High	High	Food system can adapt overall; though impacts would be felt by some rural and relatively food-insecure groups. Agricultural expansion may occur, the hunting industry – and revenues generated for conservation – would suffer large economic losses. Social cost for recreational hunters would be high.	^32^
**China**	Moderate overall, high in some areas	Moderate	High	Food system can adapt overall, though increases in agricultural production or imports would be needed, with risks for biodiversity and EIDs. Significant economic shocks for rural wildlife farmers.	33, 34, 35
**Nigeria**	High in rural areas	High	Moderate	Food system could potentially adapt through expansion of animal agriculture and provision of alternatives to rural communities, though with concomitant risks for biodiversity and EIDs. Taste preferences for wild meat over domestic meat would remain challenging, though public health messaging may overcome this.	36, 37, 38

Shading corresponds to type of negative consequences that are more likely, as per the spectrum in the conceptual model (see Methods): food insecurity = yellow, land-use change and biodiversity loss = blue. The categoric measures of ecological and socio-economic resilience and adaptability are semiquantitative, based on expert judgement by the authors. See Table S3 for details.

In other places, however, food systems could more easily absorb or adapt to the removal of wild meat. These include places where agriculture is already high-yielding, where there are available land and favorable biotic conditions for agricultural expansion, and/or where food systems are already more diversified, and people have the capacity and willingness to adapt (e.g., China, USA, Nigeria, the Brazilian Atlantic Forest, and tropical south west Ghana, Table 1). However, where animal agriculture represents a likely replacement for wild meat, this would be associated with negative consequences for biodiversity and EID risk. For example: the continued loss and fragmentation of the Atlantic forest in Brazil, which will likely result in extinction of endemic species;^27^ and further outbreaks of swine flu, which is already devastating farmers in Nigeria^39^ and may be mutating into new strains with pandemic potential in China.^40^ In addition, if rapidly growing demand for commercial meat cannot be met by domestic agriculture (e.g., in China), imports may increase,^41^^,^^42^ thus displacing biodiversity and EID risks elsewhere. Importantly, while these food systems may be more adaptable on average, the impacts and adaptive burden would be heterogenous across groups and households, and other economic, social and cultural costs may be significant. For example, rural wildlife farmers in China and female traders in Ghana could suffer major economic shocks if wildlife markets closed, while the rights and cultural values of indigenous populations in the Brazilian Atlantic forest (and indigenous territories throughout the world) would be violated if all hunting and consumption were prohibited (Table 1, Table S3). Such groups are already vulnerable to food-system shocks, and closing wildlife markets may remove an important socio-economic and nutritional safety net. Even in countries with high-yielding food systems, like the USA, access to other forms of animal protein and nutrients would need to expand for rural and marginalised communities that are relatively more dependent on wildlife.^32^ The social costs for recreational hunters in the USA, and the economic cost to conservation organizations that rely on hunting permits for income, would also be significant and difficult to replace. The contrasting outlooks for two regions in Brazil (the tropical Amazon and the Atlantic Forest) highlights the heterogeneity of wildlife use within countries, demonstrating how the resilience and adaptability of food systems vary with socioeconomic and biological context, cultural practices and landscape features and enforcement dissonance (Table 1, Table S3). All of these factors should be considered when designing policy interventions in wildlife markets.

## Discussion

Calls for prohibitions on wildlife use and trade are motivated by the desire to protect public health and biodiversity. However, our analyses reveal that overly stringent policies risk negative consequences for food security, biodiversity and public health, due to displacement and trade-offs within the broader food system. Appropriate policy formulation must consider equity issues and the rights of indigenous and tribal peoples; be informed by place-specific understandings of food systems and their adaptive capacities; and weigh-up the entire range of costs and benefits of different policy scenarios, including potential displacement of food system impacts.

### Acknowledging inequity

As our results show, some of the world’s least developed countries (e.g., Côte d’Ivoire, Madagascar, Republic of Congo; Figure 1, Figure S1) are those which are at greatest risk of negative consequences from prohibitions on wild meat. Fragile food systems would struggle to absorb or adapt to loss of wild meat from diets. This could intensify chronic health issues driven by malnutrition, such as stunted growth and impaired cognitive function, with further burdens on society,^43^, ^44^, ^45^, ^46^ or create severe trade-offs between food security and conservation (Figure 1). These consequences render complete prohibitions impractical or unacceptable in many countries: prohibitions could do more harm than good and raise serious ethical questions regarding the structural inequalities of global wildlife protection.^47^

Importantly, negative consequences would not be uniform within nations (Table 1). Indigenous, rural and socially marginalized groups may be most severely impacted, which could create and accentuate inequalities.^32^^,^^48^^,^^49^ Even in food-secure developed nations like the USA and Canada, which in principle can absorb or adapt to a shock, some marginalized groups, such as migrant and seasonal workers and rural communities, would be impacted nutritionally, economically and culturally.^32^^,^^50^ In contrast, some groups, such as wealthy urban populations who consume wildlife as a luxury good,^32^^,^^48^ may find it easier to adapt. Additional inequities—beyond the food systems impacts we explore here—include the loss of livelihoods, rights and social values, which may also undermine incentives for sustainable use.^30^^,^^32^^,^^33^^,^^51^, ^52^, ^53^

Risk-based regulation could be a more practical and socially just approach: preventing the use and trade of slowly reproducing, endangered species, or those with high zoonotic potential (e.g., great apes and bats)^54^ while permitting use and trade of faster-growing species with high potential for sustainable management and minimal public health risks (e.g., cane rats, some amphibians, and reptiles).^55^ For example, in Amazonia, there are instances of well-regulated subsistence hunting that support biodiversity conservation and human well-being^13^ and provide cost-effective strategies to control zoonoses by empowering households and communities to assume responsibility for disease control.^56^ In rural Nigeria and China, small-scale farming of low-disease-risk species such as reptiles, amphibians, and cane rats could provide sustainable protein sources, which satisfy local taste preferences, and have lower biodiversity loss and EID risks than conventional domestic livestock.^36^^,^^57^, ^58^, ^59^ In some cases, it may be feasible to substitute wild meat with other forms of plant or animal protein; however, such efforts must be sustainable, respect the customs and capacities of affected people, and avoid further habitat degradation and EID risks through expanding human-wildlife-livestock interfaces.^10^^,^^14^^,^^60^ Affected communities should also be included in decision-making, for practical, ethical, and legal reasons.^19^^,^^61^

### A food systems approach

Risk-based regulation of wildlife use and trade would benefit from better data on wild meat consumption patterns, and the feasibility of substitutes. For example, more than 100 countries were not included in this study due to missing data. Notable omissions include Sierra Leone, Gabon, DR Congo and Uganda, which have been identified as wild meat consumption hotspots in previous local-scale studies.^23^^,^^62^ The data also include some notable anomalies. For example, Russia has a long history of recreational and food-motivated hunting,^63^^,^^64^ yet has very low reported domestic consumption (320 kg) in FAO food balance sheets (Figure 1; Table S1). Similarly, several countries in South East Asia (e.g., Indonesia and Malaysia) have large, widespread wildlife markets,^65^ yet have zero “game meat” consumption in the GENuS database and FAO food balance sheet. Finally, even where data is available, it may be far below the “true” consumption, due to widespread informal and unmonitored trade networks. For example, we estimate Brazil’s national consumption as 16,250,000 kg per annum (Figure 1; Table S1), yet previous studies have estimated that consumption in Amazonia alone may be five times this mass.^66^ These omissions and anomalies likely represent inconsistencies in reporting categories and reporting effort. We acknowledge that the datasets used in this study rely on government reporting, and since wild meat is typically an informal sector, consumption will be under-reported, particularly in less developed countries where monitoring is less stringent (and wild meat is often most important). As such, we likely underestimate the food insecurity and land-use change impacts of removing wild meat from global food systems. Future analyses could benefit from broader geographic and demographic coverage of detailed wildlife use surveys (e.g.,^48^), or methods to correct for monitoring and reporting bias, such as those that have been applied to ivory seizures.^67^

It is also possible that fisheries and aquaculture could substitute for wild meat in some areas;^68^ or that increases in yield rather than expansion could help to meet demand for animal agricultural, both of which would buffer any biodiversity impacts of a wild meat ban.^69^ However, it’s unlikely that these represent viable solutions within the rapid time frame that bans on wild meat consumption could take place. The majority of global fish stocks are fished at or over capacity, while falls in fish catches are already threatening food security in low latitude developing nations—many of which overlap with the high-risk nations identified in this analysis.^70^, ^71^, ^72^, ^73^ Aquaculture can also have significant environmental and social impacts,^74^^,^^75^ and few countries currently have the technology, infrastructure and capacity to rapidly and sustainably scale-up aquaculture to replace wild meat where it is most needed.^14^ Similarly, while there have been examples of rapid agricultural yield increases at the national level in some countries, these require coordinated investment in agricultural extension, resources, infrastructure and education. Historical trends demonstrate that the norm is for yields to increase linearly,^76^ and in many of the countries and regions where the impacts of a wild meat ban are likely to be most severe, these increases are very slow indeed.^69^ Cultural uptake will also influence the success of these alternatives, such that a better understanding of the place-specific feasibility of fisheries, aquaculture and rapid yield increases, as more sustainable substitutes for wild meat, are needed to guide future interventions.^77^ Undoubtedly, wild-meat consumers in some places will face similar issues with converting to agricultural production/adopting domestic meat, and in the absence of other feasible alternatives, may face nutritional shortfalls, or inability to comply with regulations leading to a business-as-usual scenario.

By highlighting the potential negative consequences of widespread prohibitions of wild meat trade and consumption, we urge decision-makers to adopt a risk-based approach to managing wildlife use in response to COVID-19; one which considers all the costs and benefits of wildlife trade - and proposed regulations - on a case-by-case basis.^55^^,^^77^ A more holistic approach - implemented via targeted disease mitigation at critical control points throughout all human and animal interactions (including animal agriculture)^78^ - could help to reduce the risk of future pandemics and conserve wild biodiversity without such widespread negative consequences. Importantly, due consideration should also be given to the broader macro-economic shocks caused by COVID-19, and how these will influence wildlife markets and food systems.^24^ Global food systems may become less resilient due to impacts on supply chains and agricultural production, which may increase reliance on wild meat as a safety net in some areas, and potentially increase the negative consequences of prohibiting its consumption. Policy responses to COVID-19 should be holistic and future-proof, to ensure they support recovery from the current social and economic crisis, and set the world on a pathway to sustainability.

## STAR★Methods

### Key resources table

**Table:** 

REAGENT or RESOURCE	SOURCE	IDENTIFIER
**Deposited Data**		

Global Expanded Nutrient Supply (GENuS) database: Nutrient Supplies by Food and Country	^7^	https://doi.org/10.7910/DVN/UZW5S3, Table S1
FAO food balance sheet data	^4^^,^^79^	Table S1
The Economist Global Food Security Index (GFSI)	^9^^,^^80^	Table S1
Country-specific Characterization Factors for land use impacts on biodiversity	^8^	https://doi.org/10.1021/acs.est.5b02507,
Region- and livestock-specific estimates of land demand per gram of protein based on life-cycle assessments	^11^	https://doi.org/10.1126/science.aaq0216,
All code used for the analysis (deposited in Zenodo)	^22^	https://doi.org/10.5281/zenodo.4005563
National-level wild meat consumption estimates	This paper	Table S1
National-level land demand estimates	This paper	Table S2
National-level biodiversity loss estimates	This paper	Table S2

### Resource availability

#### Lead contact

Requests for further information will be fulfilled by the Lead Contact: Hollie Booth (hollie.booth@zoo.ox.ac.uk).

#### Materials availability

This study did not generate new, unique reagents.

#### Data and code availability

The datasets and code used and generated during this study are available in the Supplemental Information and at the following Zenodo repository: https://zenodo.org/record/4415553.

### Experimental model and subject details

The primary study subject was national-level nutrient supply from food, which is available for 23 individual nutrients across 225 food categories in the GENuS database.^7^^,^^81^ This dataset is prepared as per the methods outlined in Smith et al. (2016), and maintained by the University of Harvard Chan School of Public Health. We supplemented gaps in the GENuS database with additional data from FAO food balance sheets and a recently compiled dataset of bushmeat consumption,^4^^,^^79^ which provide government-reported supplies of food items available for human consumption, along with their caloric value and protein and fat content. This dataset is prepared and maintained by the Food and Agriculture Organization of the United Nations (FAO). Other data used in the analysis include the Global Food Security Index (GFSI), which is a quantitative and qualitative benchmarking model that measures drivers of food security across 113 countries,^80^ and is prepared and maintained by The Economist Intelligence Unit; country-specific characterization factors of biodiversity loss from conversion of natural habitats to agricultural land, as calculated and published in Chaudhary et al. (2015);^8^ and region- and livestock-specific estimates of land demand per gram of protein based on life-cycle assessments, as calculated and published in Poore and Nemecek (2018).^11^

### Method details

#### Conceptual framework

**Figure 2:**
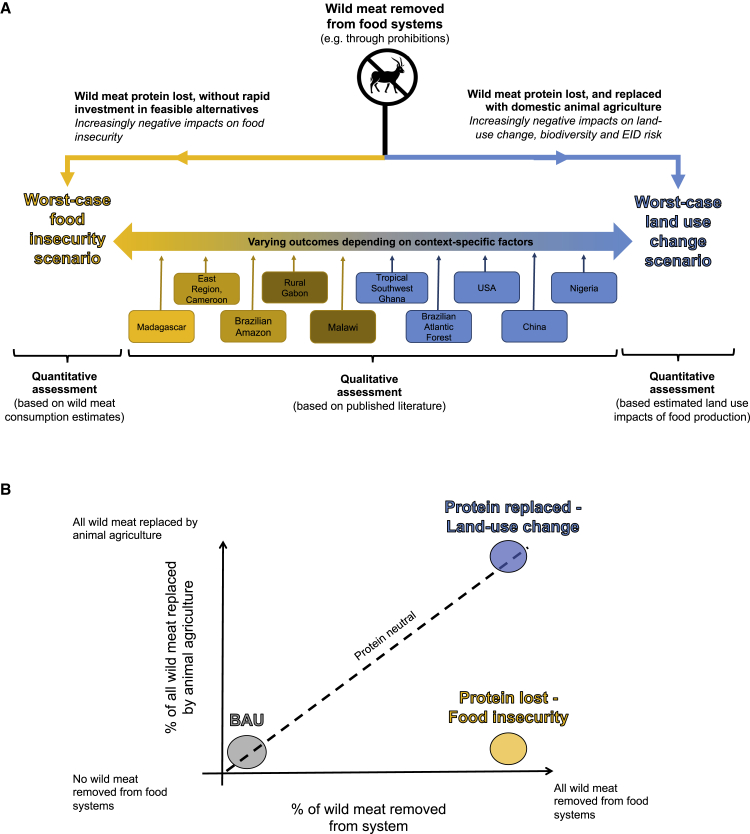
The conceptual framework for this study: a spectrum of negative consequences, and the methods used to assess them We note that the negative consequences depicted in (A) interact and are inter-dependent, as shown in (B), such that increasing removal of wild meat requires increasing land-use change for animal agriculture in order to maintain current levels of protein. The protein neutral line assumes complete, direct substitution of protein between wild meat sources and animal agriculture source.

The potential negative consequences of a policy-induced loss of wild meat from food systems exist on a spectrum between two ‘worst-case scenarios’ (Figure 2). A worst-case scenario for food insecurity is one in which all wild meat is suddenly lost from food systems, in the absence of feasible, socially desirable alternatives, meaning that the protein is not replaced (Figure 2). Conversely, if all wild meat is replaced by animal agriculture, this could lead to a worst-case land-use change scenario, with subsequent impacts on biodiversity and EID risk. Alternatively, a lack of enforcement or social acceptance of policies to restrict wild meat supply could result in a business as usual (BAU) scenario, where prohibitions have little effect. Prohibitions can also lead to other perverse consequences, such as proliferation of informal and illicit trade networks, which undermines evidence-based surveillance and disease mitigation, and may increase prices and fuel further corruption and inequity in places where enforcement capacity is weak.^19^^,^^82^ In reality, consequences would likely fall somewhere in between these three extremes (Figure 2), moderated by levels of compliance and modes of adaptation (e.g., adoption of less-damaging alternatives such as wild-caught fisheries, aquaculture, small-mammal farming, sustainable wildlife hunting or cheap food imports), which in turn depend on system-specific socio-ecological factors, such as culture and biomes.^14^^,^^19^^,^^83^

We used a mixed-methods approach to explore potential negative consequences along this spectrum. We first use the two contrasting “worst-case” scenarios to quantitively explore global patterns in the potential magnitude of negative consequences. We build on early attempts by Fa et al. (2003) to quantify linkages between wild meat and food security, using recently assembled global datasets on protein supply, food security, land-use change and biodiversity loss from different types of agriculture.^4^^,^^7^, ^8^, ^9^, ^10^, ^11^ We estimate the animal protein that would be lost from diets, should all wild meat be removed from diets, and the land required to replace this protein with livestock production in 83 countries. We acknowledge that these scenarios are unlikely to occur in full, but use them to highlight which countries could face the largest shocks from the loss of wild meat in food systems. We then qualitatively explore how context-specific idiosyncrasies might plausibly affect the consequences of prohibitions on wildlife trade in 10 case study places, that represent a range and diversity of possible outcomes (Figure 2): Madagascar; East Region Cameroon; the Brazilian Amazon; rural Gabon; Malawi; tropical Southwest Ghana; the Brazilian Atlantic Forest; USA; China and Nigeria.

#### Quantitative assessment

To assess the impacts of the removal of wild meat from food systems we focused on protein as an indicator for the range of important micro- and macro-nutrients sourced from animal meat.^84^ We acknowledge that a range of other important nutrients, vitamins and fatty acids are sourced from animal meat,; however, all of these nutrients will scale in proportion with mass consumed, therefore the overall patterns will be similar. We first estimated annual wild meat consumption for every country for which data were available. We based our estimates on the GENuS database^7^ and calculated total annual consumption in a country by multiplying consumption of wild meat protein per person per day as (W_PPPD_, Table 2; Smith, 2016) by the total population of the country in 2019.^85^ We assumed all wild meat was categorised as ‘game meat’ in the GENuS database, though acknowledge this may underestimate wild meat consumption as it may not capture some types of wildlife consumed for food (e.g., wildfowl, farmed reptiles and amphibians), and reporting biases will vary by country, with underreporting likely in places where wild meat is an informal sector. We supplemented these data with additional data from FAO food balance sheets and a recently compiled dataset of bushmeat consumption.^4^ For FAO data we calculated the consumption of ‘game meat’ as the trade balance (imports minus exports) plus the national annual production. For these datasets we further had to convert live-weight into protein, basing calculations on another recently published dataset.^11^ In total, this resulted in 83 countries with non-zero estimates of wild meat consumption (Table S1). We acknowledge that these datasets are imperfect, and likely represent conservative estimates of wild meat consumption: they rely on government reporting, yet wild meat is often traded and consumed within informal and subsistence markets, which are likely to be un-reported, particularly in countries where monitoring is less stringent. Nonetheless, they represent the best-available data for a rudimentary global analysis of this important yet overlooked issue.

**Table 2:** Summary of all calculations used in quantitative assessment of impacts on food security and land use

Equation 1. Current levels of wild meat consumption							
Total annual wild meat consumption per country per annum (**W)**	=	**Equation**	Daily protein (g) per person per day from game meat (W_PPPD_)	X	National population estimate	X	365.25
		**Data source**	GENuS database ^7^		UN 2019 population estimates^82^		Days per year

Equation 2. Hypothetical protein consumption if under worst-case food insecurity scenario							
Hypothetical protein deficit if wild meat is removed without alternatives (**P**_**removal**_)	=	**Equation**	Total protein intake per person per day from all foods (P_current_)	-	W_PPPD_		
		**Data source**	GENuS database (Smith, 2016)		GENuS database ^7^		

Equation 3. Hypothetical land demand under worst-case land use change scenario					
Hypothetical land use change (km^2^) if all wild meat protein is replaced with animal agriculture (**L**_**demand**_)	=	**Equation**	W	X	$$\left(\begin{array}{lll} \text{Pasture}\;\text{demand}\;\text{per}\;\text{unit}\;\text{of}\;\text{meat}\;\text{replacement} & & \text{Cropland}\;\text{demand}\;\text{per}\;\text{unit}\;\text{of}\;\text{meat}\;\text{replacement}\\ \sum_{i-1}^{n}Lpas & + & \sum_{i-1}^{n}Lcrop_{i} \end{array}\right)$$
		**Data source**	Equation 1		Where *i* is the different livestock sectors within a country (beef, sheep/goat, pork, poultry), weighted according to current consumption levels (estimated from Smith (2016)), and L is land needed per sector (km^2^/kg) based on region-specific estimates of land demand per unit of protein, for pasture (Lpast) and cropland for feed (Lcrop)^11^

Equation 4. Hypothetical biodiversity loss under worst-case land use change scenario			
Hypothetical biodiversity loss (no. species) if all wild meat protein is replaced with animal agriculture (**B**_**loss**_)	=	**Equation**	$$\left(\begin{array}{lll} {\text{L}}_{\text{demand}}\left(\text{pasture}\right) & \text{X} & \left(\text{Cpast}+\left(10\;\text{x}\;\text{Opast}\right)\right) \end{array}\right)+\left(\begin{array}{lll} {\text{L}}_{\text{demand}}\left(\text{crop}\right) & \text{X} & \left(\text{Ccrop}+\left(10\;\text{x}\;\text{Opast}\right)\right) \end{array}\right)$$
		**Data**	Where L_demand_ (pasture) is the land demand component for pasture only and L_demand_ (Crop) is the land demand component for crop only (Equation 3). **C** and **O** are country-specific “global characterization factors” of the number of species destined for extinction, caused by: **C** = one-off impact of conversion of natural habitats to pasture (**C**past) and cropland (**C**crop). **O** = ongoing impact of land occupation (assumed to be 10 years in this study) by pasture (**O**past) and cropland (**O**crop)^8^

#### Food insecurity scenario

To identify countries where loss of wild meat protein could have negative impacts on food security, we plotted consumption of wild meat protein per person per day (W_PPPD_, Table 2) against global food insecurity rank^9^ for 54 countries with data available for both wild meat consumption and food insecurity. Daily per capita consumption of wild meat protein indicates the magnitude of the shock a country’s food system might face if wild meat were suddenly removed, while food insecurity rank provides an indication of how robust each country’s food systems currently are. For each country, we also estimated hypothetical per capita protein intake in the absence of wild meat with no alternatives (P_**removal**_) (i.e., the worst-case food insecurity scenario) as per Equation 2 (Table 2), and identified countries where P_**removal**_ falls below recommended healthy intakes of protein according to the World Health Organization.^84^ This indicates which countries may face severe protein deficits, though many countries currently consume in excess of the WHO recommended daily intake of protein, and could feasibly reduce protein intake against current levels without major impacts on nutritional security.

#### Land-use change scenario

To estimate the worst-case land-use change (L_demand_), we first estimated the production of domestic livestock (beef, sheep/goat, pork, poultry) required to replace all wild meat protein (W), based on their current share of consumption in the country (Equation 3, Table 2). For example, if meat from poultry, beef, and pigs respectively accounted for 20%, 30%, and 50% of a country’s current protein consumption from meat, then 20% of wild meat protein would be replaced by protein from poultry, 30% by protein from beef, and 50% by protein from pigs. We then estimated the additional land needed to support the additional production using region- and livestock-specific estimates of land demand per gram of protein, including both pasture and cropland for feed production.^11^ We then summed across livestock species to provide an estimate of the total additional agricultural land that would be required in each country (Equation 3, Table 2). Where region-specific land-demand estimates were lacking, we used global estimates. To investigate the potential negative consequences of this land-use change on biodiversity (B_loss_), we used country-specific characterization factors of biodiversity loss from conversion of natural habitats to agricultural land.^8^ These characterization factors are reported as the number of species destined to become extinct from agricultural activities in the long-term per unit area. These factors are based on Countryside Species-Area Relationships,^20^ and species richness and endemism in different countries, and the affinity of different taxonomic groups for different land uses as calculated by Chaudhary et al.^8^ Data limitations mean that characterization factors are limited to four groups of terrestrial vertebrates (amphibians, birds, mammals and reptiles), so we likely under-estimate total extinctions likely to result from land-use change and occupation because we do not include other taxa such as plants or insects in our analysis. Separate estimates have been calculated for cropland or pasture, as well as separate estimates of the one-off biodiversity impact of land-cover change and of the annual biodiversity impact of continued occupation and production on cropland or pasture.^8^ For each country, we calculated the total biodiversity impact as the sum of the one-off impact of the land-cover transition (calculated as estimated additional cropland (pasture) multiplied by the characterization factor for conversion into cropland (pasture)) and the on-going impact of land occupation over a 10-year period (calculated as estimated additional cropland/pasture multiplied by the characterization factor for the annual impact of production on cropland (pasture); see also Equation 4, Table 2). This likely represents a conservative estimate of the impacts of land-use change as on-going biodiversity loss is likely to continue for longer (e.g., Hendershot et al., 2020). We also consider the impact this land-use change could have on EID risk, since degree of land-use change is known to be a key predictor of EID events.^10^

#### Qualitative assessment

These analyses provide plausible bounds for the impacts of a reduction in wild meat consumption, but the actual responses of food systems will be idiosyncratic and shaped by local and national context. To explore how context could shape responses, we outline plausible narratives for how policy-induced removal of wild meat (i.e., prohibitions on wildlife trade and consumption) might impact food systems in 10 case studies. We qualitatively investigate drivers of wild meat consumption and overall food system adaptability, considering current levels of consumption and dependence on wild meat, and environmental and socio-economic factors that influence food system resilience and adaptability, e.g., land availability for agricultural expansion; seasonality of agriculture; technological and human capacity; relative price of and access to alternative protein sources; the degree of urbanization and proximity to wildlife; wealth, cultural preferences and willingness to change consumption patterns; and the perceived legitimacy of regulations.^19^^,^^48^^,^^83^

### Quantification and statistical analysis

We conducted all quantitative analysis using RStudio,^21^ the code has been made publicly available via Zenodo.^22^ We did not conduct any statistical analysis in this study.

## Supplemental information

Document S1. Figure S1 and Tables S1–S3

Document S2. Article plus supplemental information
