# Supplementary material for: Investigating the risks of removing wild meat from global food systems
Source: Curr Biol. 2021 Apr 26;31(8):1788–1797.e3. doi: 10.1016/j.cub.2021.01.079 (PMC8094154; doi:10.1016/j.cub.2021.01.079)
Supplement: Document S1. Figure S1 and Tables S1–S3 [file mmc1.pdf]

Current Biology, Volume *31*

Supplemental Information

Investigating the risks of removing wild meat from global food systems

Hollie Booth, Michael Clark, E.J. Milner-Gulland, Kofi Amponsah-Mensah, André Pinassi Antunes, Stephanie Brittain, Luciana C. Castilho, João Vitor Campos-Silva, Pedro de Araujo Lima Constantino, Yuhan Li, Lessah Mandoloma, Lotanna Micah Nneji, Donald Midoko Iponga, Boyson Moyo, James McNamara, O. Sarobidy Rakotonarivo, Jianbin Shi, Cédric Thibaut Kamogne Tagne, Julia van Velden, and David R. Williams

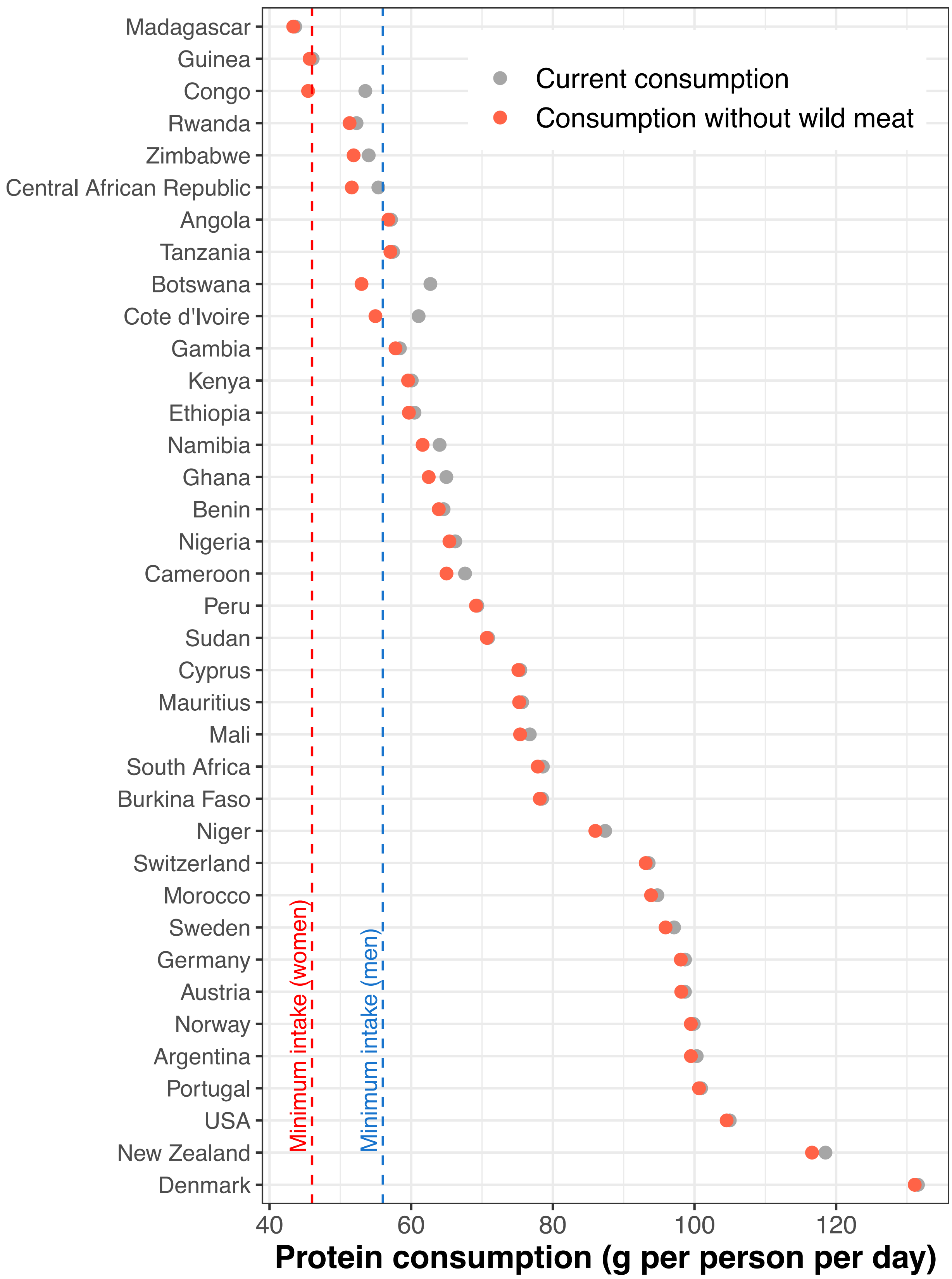

Figure S1. Estimated per capita protein deficits caused by loss of wild meat from diets, in the absence of replacements (current estimated total protein intake minus estimated game meat protein intake). Minimum protein intake values based on guidelines from the World Health Organisation. Relates to Figure 1.

COUNTRY	ISO3	Protein from game meat per person per day - GENUS (g)	Total annual protein from game meat - GENUS (kg)	Protein from all meat per person per day - GENUS (g)	Total annual protein from all meat - GENUS (kg)	Percent protein from game meat	Protein per person per day without game meat (g)	Game meat data source for analysis	Global Food Security Index
Nigeria	NGA	0.83	62,254,085	3.55	267,314,892	23.3	65.40	GENUS	94
Cote d'Ivoire	CIV	6.10	58,766,637	8.31	80,057,453	73.4	54.95	GENUS	84
USA	USA	0.44	53,679,031	34.01	4,111,466,970	1.3	104.54	GENUS	3
Ethiopia	ETH	0.79	33,254,337	2.85	119,751,625	27.8	59.68	GENUS	91
Ghana	GHA	2.49	28,296,302	7.16	81,286,399	34.8	62.48	GENUS	59
Cameroon	CMR	2.62	25,383,805	6.50	63,070,135	40.2	64.99	GENUS	88
Germany	DEU	0.60	18,265,160	24.19	740,156,031	2.5	98.07	GENUS	11
Congo	COG	8.08	16,289,926	17.28	34,827,648	46.8	45.44	GENUS	
South Africa	ZAF	0.72	15,499,301	19.56	423,707,390	3.7	77.87	GENUS	48
Argentina	ARG	0.82	13,602,721	32.83	541,958,029	2.5	99.50	GENUS	37
Niger	NER	1.39	12,264,897	5.75	50,878,149	24.1	86.00	GENUS	89
Morocco	MAR	0.89	12,042,828	11.33	152,739,431	7.9	93.87	GENUS	59
Zimbabwe	ZWE	2.13	11,582,638	7.79	42,302,051	27.4	51.87	GENUS	
Mali	MLI	1.37	10,127,448	8.57	63,418,153	16.0	75.38	GENUS	80
Kenya	KEN	0.52	10,119,492	4.68	91,968,972	11.0	59.57	GENUS	86
Botswa	BWA	9.72	8,352,768	15.86	13,619,480	61.3	52.99	GENUS	57
Tanzania	TZA	0.38	8,232,121	3.23	70,448,317	11.7	57.06	GENUS	96
CAR	CAF	3.76	6,624,268	13.91	24,536,024	27.0	51.61	GENUS	
Rwanda	RWA	0.99	4,667,121	2.58	12,217,856	38.2	51.30	GENUS	95
Sweden	SWE	1.20	4,432,779	22.73	83,839,357	5.3	95.92	GENUS	7
Angola	AGO	0.37	4,409,458	10.29	123,542,743	3.6	56.79	GENUS	100
New Zealand	NZL	1.90	3,347,918	38.78	68,308,487	4.9	116.60	GENUS	19
Iran	IRN	0.11	3,221,848	11.29	346,209,703	0.9	101.57	GENUS	
Benin	BEN	0.68	3,016,751	6.38	28,239,351	10.7	63.89	GENUS	85
Sudan (former)	SDN	0.19	3,001,548	7.04	112,774,175	2.7	70.68	GENUS	99
Madagascar	MDG	0.28	2,797,673	4.61	46,657,906	6.0	43.35	GENUS	108
Burki Faso	BFA	0.33	2,544,337	5.17	39,506,942	6.4	78.16	GENUS	87
Peru	PER	0.19	2,340,912	6.91	83,177,886	2.8	69.15	GENUS	58
mibia	M	2.40	2,230,420	12.53	11,632,901	19.2	61.61	GENUS	
Guinea	GIN	0.43	2,075,939	2.87	13,750,757	15.1	45.66	GENUS	97
Austria	AUT	0.52	1,714,895	28.31	93,140,068	1.8	98.13	GENUS	10
Chi	CHN	0.00	1,711,143	15.79	8,300,536,782	0.0	90.27	GENUS	35
Switzerland	CHE	0.42	1,334,269	20.92	66,121,410	2.0	93.11	GENUS	4
United Kingdom	GBR	0.05	1,170,366	24.35	603,768,499	0.2	91.38	GENUS	17
France	FRA	0.05	1,129,570	25.52	608,339,578	0.2	112.11	GENUS	16
Portugal	PRT	0.29	1,081,960	25.11	93,531,745	1.2	100.64	GENUS	20
Denmark	DNK	0.46	971,146	22.19	46,947,588	2.1	131.09	GENUS	14
Spain	ESP	0.06	962,316	25.95	443,068,592	0.2	95.10	GENUS	25
Italy	ITA	0.04	896,112	24.91	550,031,244	0.2	106.46	GENUS	23
Romania	ROU	0.13	892,374	14.93	104,892,909	0.9	97.39	GENUS	38
Poland	POL	0.06	859,378	20.24	279,839,753	0.3	100.54	GENUS	24

Norway	NOR	0.41	815,329	18.96	37,546,579	2.2	99.49	GENUS	5
Netherlands	NLD	0.11	668,847	23.98	150,102,282	0.4	126.77	GENUS	9
Gambia	GMB	0.61	542,376	3.11	2,747,366	19.7	57.79	GENUS	
Belgium	BEL	0.10	430,253	21.50	91,008,519	0.5	102.10	GENUS	15
Mauritius	MUS	0.42	195,792	17.52	8,136,858	2.4	75.25	GENUS	
Slovakia	SVK	0.08	169,304	17.03	33,950,672	0.5	73.98	GENUS	47
Czech Republic	CZE	0.04	151,162	23.15	90,538,968	0.2	86.36	GENUS	32
Uruguay	URY	0.10	127,309	22.95	29,122,487	0.4	106.62	GENUS	33
Cyprus	CYP	0.28	124,848	22.96	10,126,283	1.2	75.13	GENUS	
Tunisia	TUN	0.03	117,411	8.37	36,133,443	0.3	94.18	GENUS	69
Finland	FIN	0.05	105,026	20.63	41,743,592	0.3	107.21	GENUS	5
Greece	GRC	0.02	65,717	23.19	88,267,742	0.1	115.18	GENUS	31
Luxembourg	LUX	0.24	54,537	27.58	6,305,412	0.9	110.09	GENUS	
Ireland	IRL	0.03	52,479	22.36	40,323,527	0.1	135.51	GENUS	2
Senegal	SEN	0.00	25,600	4.71	28,811,554	0.1	57.21	GENUS	81
Lithuania	LTU	0.02	22,689	20.05	19,935,347	0.1	131.22	GENUS	
Slovenia	SVN	0.02	14,360	22.42	17,020,490	0.1	89.64	GENUS	
Kazakhstan	KAZ	0.00	10,383	21.71	148,906,158	0.0	90.42	GENUS	48
UAE	ARE	0.00	8,964	16.89	61,030,837	0.0	98.34	GENUS	21
Bulgaria	BGR	0.00	5,302	16.31	41,393,014	0.0	78.60	GENUS	51
Malta	MLT	0.01	1,615	25.02	4,034,812	0.0	98.95	GENUS	
Russian Federation	RUS	0.00	677	19.62	1,045,774,988	0.0	92.85	GENUS	42
Ecuador	ECU	-	16,250,000	17.58	113,282,734	-	71.48	Halpern et al 20	63
Georgia	GEO	-	6,500	7.83	11,415,074	-	97.50	FAO	
Bahamas	BHS	-	2,080	28.55	4,100,189	-	63.39	FAO	
Indonesia	IDN	-	1,950	3.81	380,485,161	-	59.16	FAO	62
Cabo Verde	CPV	-	1,430	15.24	3,094,569	-	69.69	FAO	
Albania	ALB	-	260	12.88	13,542,886	-	94.09	FAO	
Guya	GUY		16,250,000	13.04	3,746,473	433.7		Halpern et al 2019	
Surime	SUR		16,250,000	17.58	3,767,034	431.4		Halpern et al 2019	
Bolivia	BOL		16,250,000	21.52	91,753,350	17.7		Halpern et al 20	75
Colombia	COL		16,250,000	15.19	282,250,624	5.8		Halpern et al 2019	
Venezuela	VEN		16,250,000	27.93	290,112,828	5.6		Halpern et al 20	113
Brazil	BRA		16,250,000	29.55	2,294,403,831	0.7		Halpern et al 20	39
Zambia	ZMB		4,940,000	-	-			FAO	101
Gabon	GAB		3,315,390	-	-			FAO	
Afghanistan	AFG		1,040,000	-	-			FAO	
Liberia	LBR		1,040,000	-	-			FAO	
Togo	TGO		733,980	-	-			FAO	102
Lesotho	LSO		676,000	-	-			FAO	
Chad	TCD		585,000	-	-			FAO	109
Sierra Leone	SLE		396,760	-	-			FAO	106

Table S1. Estimated annual wild meat consumption and food security indices for 83 countries with non-zero estimates. Related to Figure 1.

Country	ISO3	Estimated extra pasture (km2)	Estimated extra crop land (km2)	Total estimated extra agricultural land (km2)	Estimated number of species destined for extinction
Ecuador	ECU	6082.2	1262.7	7344.9	85.1
Colombia	COL	6711.1	1284.7	7995.8	41.8
United States	USA	8473.3	3808.4	12281.7	24.8
Venezuela	VEN	4274	1110	5384.1	15.1
Cote d'Ivoire	CIV	5302.8	1532.4	6835.2	12.4
Cameroon	CMR	3104	754.2	3858.2	10
Brazil	BRA	7857.2	1380.8	9238	8.2
Nigeria	NGA	8617.5	1702.1	10319.6	6.3
Suriname	SUR	3853.2	1048.7	4901.9	6.2
Bolivia	BOL	5759.8	1188	6947.7	5.9
Guyana	GUY	1497.4	803.1	2300.5	5.3
Madagascar	MDG	322.8	87.6	410.5	4.8
Ghana	GHA	1556.1	657.7	2213.7	3.7
Rwanda	RWA	863.5	177.8	1041.3	3.6
Ethiopia	ETH	6351.7	1033	7384.7	3.2
Morocco	MAR	1449.4	425.1	1874.5	3.2
Argentina	ARG	4106.7	627.3	4734	2.8
New Zealand	NZL	1198.5	217.6	1416.1	2.8
South Africa	ZAF	1142	447	1589	2.5
Tanzania	TZA	1244.8	248.8	1493.6	1.9
Germany	DEU	1087.6	754.3	1841.8	1.9
Gabon	GAB	355.4	237.9	593.3	1.6
Peru	PER	263.9	65.8	329.7	1.4
Liberia	LBR	73.4	77.2	150.5	1.4
Kenya	KEN	1968.1	338.7	2306.7	1.3
Congo - Brazzaville	COG	330.9	359.5	690.4	1.3
Mauritius	MUS	9.9	5.5	15.4	0.8
China	CHN	111.2	85.2	196.4	0.7
Portugal	PRT	84.1	52	136.1	0.7
Iran	IRN	161.4	66.8	228.1	0.5
Italy	ITA	88.4	47.9	136.4	0.5
Spain	ESP	63	48	111	0.5
Zambia	ZMB	1796.5	455.4	2251.9	0.4
Austria	AUT	99.4	65.9	165.3	0.4
Sierra Leone	SLE	101.6	31.2	132.8	0.4
Zimbabwe	ZWE	1289.3	391.8	1681.1	0.4
Guinea	GIN	361.3	68.4	429.6	0.4
Switzerland	CHE	90	45	135	0.3
Central African Republic	CAF	1359	253.8	1612.8	0.3
Sweden	SWE	336.8	200.9	537.7	0.3
Lesotho	LSO	264.2	57.6	321.8	0.3
Cyprus	CYP	8.1	5.9	13.9	0.2
France	FRA	92.2	42.8	135.1	0.2
Botswana	BWA	1216.1	253.4	1469.6	0.2
Romania	ROU	68.9	47.6	116.5	0.2
Afghanistan	AFG	556.7	84.9	641.6	0.1
Togo	TGO	157.2	54.3	211.5	0.1
Namibia	NAM	250.1	66.7	316.8	0.1
Poland	POL	10.7	46	56.6	0.1
Angola	AGO	178.2	93.1	271.3	0.1
Denmark	DNK	86	40.9	126.9	0.1
Norway	NOR	176.2	77.7	253.9	0.1
United Kingdom	GBR	83.8	49.7	133.5	0.1
Mali	MLI	1725	257.7	1982.8	0.1
Benin	BEN	140.8	73	213.8	0
Niger	NER	2107.6	325	2432.7	0
Belgium	BEL	26.7	16	42.7	0
Netherlands	NLD	22.5	12.8	35.3	0
Greece	GRC	11.6	3.5	15.1	0
Burkina Faso	BFA	380.9	74.7	455.6	0
Sudan	SDN	803.8	101.9	905.7	0
Slovakia	SVK	4.5	8	12.5	0
Tunisia	TUN	18.4	4.4	22.8	0
Czechia	CZE	5	8	13	0
Gambia	GMB	39.3	12.5	51.8	0
Georgia	GEO	1.1	0.5	1.6	0

Luxembourg	LUX	8.3	4.3	12.7	0
Chad	TCD	361.1	56.3	417.4	0
Cape Verde	CPV	0.1	0.1	0.2	0
Indonesia	IDN	0.1	0.3	0.3	0
Finland	FIN	6.8	4.9	11.7	0
Slovenia	SVN	1.3	0.7	2.1	0
Bahamas	BHS	0.2	0.2	0.4	0
Uruguay	URY	12.1	2	14.2	0
Ireland	IRL	5.2	2.9	8.1	0
Lithuania	LTU	0.4	1.3	1.7	0
Bulgaria	BGR	0.2	0.3	0.5	0
Malta	MLT	0.1	0.1	0.2	0
Kazakhstan	KAZ	1.9	0.5	2.3	0
Senegal	SEN	3.3	0.7	4	0
Albania	ALB	0.1	0	0.1	0
United Arab Emirates	ARE	0.7	0.3	1	0
Russia	RUS	0.1	0	0.1	0

Table S2. Estimated land demand and biodiversity loss per country. Related to Figure 1.

Case study	Key characteristics	Key refs
**Madagascar**	**Current consumption**: High dependence on wild meat for nutrition and food security. Around Ankarafantsika National Park ~90% of households hunt wildlife at least once per week to cope with food insecurity.<br>**Environmental factors**: An island nation, with ~71% of land cultivated, and ~10% designated as protected. Other suitable forests and hillsides continue to be cleared, primarily for small-scale farming, but space for further expansion is limited. Conservation restrictions to stop forest clearance and hunting already result in significant welfare costs to communities.<br>**Socio-economic factors:** Food insecurity and malnutrition is high, poverty (as opposed to wealth) mostly drives wild meat consumption<br>**Overall assessment:** Food system would struggle to adapt to loss of wild meat - protein intake would likely be reduced for many rural households, which are already food insecure, leading to malnutrition. Alternatively, high social costs may lead to non-compliance with prohibitions, as is already the case in/around existing protected areas.	S1-4
**East Region, Cameroon**	**Current consumption**: Wild meat important for diets and nutrition, particularly in rural areas: 30-80% of protein intake in rural households<br>**Environmental factors**: Rural agriculture is subsistence and seasonal. Examples of viable alternatives to wild meat hunting in rural areas remain elusive. Small-scale aquaculture is under-developed, but requires major investment in capacity and capital, and may be unsuitable.<br>**Socio-economic factors**: Established cultural preferences for wild meat. Capacity to enforce regulations is weak in remote areas.<br>**Overall assessment**: Rural food system would struggle to adapt to loss of wild meat from diets, due to lack of space and resources for alternatives. Prohibitions may be socially illegitimate and difficult to enforce.	S5-7
**Malawi**	**Current consumption:** On average of 14% of households hunt wildlife, 21% of households consume wild meat. Hunting and consumption of wildlife is already illegal in many areas, but continues via illicit and informal markets.<br>**Environmental factors**: Agricultural production is seasonal, space for agricultural expansion is severely limited.<br>**Socio-economic factors**: Hunting is a cultural tradition in the Northern region, and there are taste preferences for wild meat over domestic meat. Households hunt for income and subsistence. Malawi is food insecure, and wild meat makes up gaps in food availability during the agricultural lean season. Supply chains for alternative protein sources are weak.<br>**Overall assessment**: Rural food system would struggle to adapt to loss of wild meat, and any additional prohibitions are likely to be socially illegitimate, with persistence of informal markets. Though urban Malawians consuming wild meat (mice and birds) as a delicacy may adapt.	S8-10
**Rural Gabon**	**Current consumption:** >70% of rural families participate in subsistence hunting, 40-60% of households sell wild meat, wild meat provides up to 90% of dietary protein in some families. Some evidence of declining hunting due to urbanisation, though there are peaks during seasonal employment gaps, and rural people remain highly dependent on forest products.<br>**Environmental factors**: Agricultural production is small-scale and seasonal<br>**Socio-economic factors**: Wild meat is a deeply-rooted cultural preference with inelastic demand. Relatively wealthier households (even in poor rural areas) consume more, and people are willing to pay more for wild meat than livestock. Hunting can make up one quarter of household income in some areas, and remote villages have low capacity to change livelihood strategies, with ability to adapt depending on proximity to facilities and infrastructure, and availability of resources.<br>**Overall assessment**: Rural food system would struggle to adapt to loss of wild meat from diets and livelihoods. Though urbanisation may reduce participation in hunting, demand for wild meat may remain or increase due to increased wealth and cultural preferences. Prohibitions may be socially illegitimate and difficult to enforce, even with livestock alternatives.	S11-15
**Brazilian Amazon**	**Current consumption:** Subsistence hunting (and fishing) is an important cultural activity and major source of dietary protein for indigenous and rural communities in remote areas of Amazonia. Estimated that 89,000 tons of wild meat are consumed per year by 8 million peoples in Brazilian Amazonia. Wild meat provides 8-72% of total protein consumed by Amazonian people, depending on socio-ecological systems. In urban centres of the interior of Amazonia, > 80% of households consume wild meat.<br>**Environmental factors:** Well-established large-scale agriculture has led to high rates of deforestation. 44% of remaining natural habitat is protected, in to which large-scale agriculture cannot expand (though could feasibly expand only ~20% of non-protected forest remnants).<br>**Socio-economic factors:** Well-established large-scale agriculture and cattle ranching in the Amazonian deforestation frontier. Market is aimed at national and international consumers, and does not supply remote rural and indigenous communities. Although hunting is permitted for indigenous peoples, uncertain legal status of hunting leaves other rural populations subject to arbitrary interpretation and weak enforcement of contradictory laws, contributing to informality and illicit markets. In urban centers prices of wild meat, chicken, and beef vary according to availability and distance to productions areas. Limited evidence that	S16-19

	incentives and social marketing can encourage alternatives (chicken) in urban centres. However, livestock raising for food provision is not common amongst rural and indigenous communities, with many previous husbandry initiatives failing for logistical, technical, social and environmental reasons.<br>**Overall assessment**: Rural and indigenous food system would be unable to rapidly adapt to loss of wild meat, primarily due to poor access to alternatives, but also due to cultural importance of wild meat. Communities would likely rely even more on fishing, since it is the most complementary protein source in most of Amazonia. Agricultural expansion may occur to increase protein supply to urban consumers. The social costs in terms of lost rights and traditions would be high, and prohibitions would be difficult to enforce. Community-based sustainable hunting of certain low-disease-risk species may represent a more viable and socially-just option.	
**Brazilian Atlantic Forest**	**Current consumption:** Mostly rural communities who hunt wildlife for diet complementation (not strictly subsistence), recreation, retaliation and trade to urban areas. In Southern Bahian ~50% of rural households hunt occasionally in protected areas primarily for consumption. Subsistence hunting is an important cultural activity and a source of animal protein for ~ 167000 indigenous. Sport and commercial hunting are also performed by urban residents. Hunting is already illegal except for satisfying hunger of a person and for indigenous peoples in officially recognized territories, though enforcement is limited, so illegal hunting continues including in strictly protected areas.<br>**Environmental factors:** Well-established agricultural sector (which could potentially intensify production) and urbanisation. A biodiversity hotspot where ~28% of original vegetation cover remains (highly dispersed and fragmented), and is under continued pressure from hunting, logging and agricultural expansion. Only ~30% of remaining forest is protected.<br>**Socio-economic factors:** Small-scale agriculture and animal husbandry are common in rural areas. Intense urbanization and access to markets mean most people can access alternative protein sources. However, cultural aspects and taste preference for wild meat are high in some areas.<br>**Overall assessment**: The food system could potentially adapt to removal of wild meat. However investments would be necessary to sustainably intensify current production and/or recover degraded areas to expand agriculture, so avoiding further deforestation and threats to biodiversity in this already highly fragmented region. The social costs would be high for the rural poor and for indigenous populations also in terms of lost rights and traditions. Existing prohibitions are already difficult to enforce, additional regulations (affecting the most vulnerable populations) may be socially unjust and result in non-compliance.	S20-22
**Tropical south west Ghana**	**Current consumption:** In rural areas ~44% of households consume wild meat on a weekly basis and ~40% engage in hunting (though not as a key livelihood). In urban areas, ~69% of people report eating wild meat, though few (6%) on a daily basis. The importance of wild meat for consumers and hunters appears to be declining, though remains an important commodity for some, particularly the rural poor.<br>**Environmental factors:** Scope for agricultural expansion into primary forests may be limited due to already highly fragmented habitat. Any increased livestock production will intensify competition in the existing agricultural landscape, with potential for escalating conflict between herders and farmers with severe social and economic consequences.<br>**Socio-economic factors:** Consumer surveys suggest preferences for wild meat are declining in urban areas, though remains an important, high-value cultural commodity. Hunting and trade is an important economic activity - it serves a safety net function during seasonal periods of economic hardship, and those involved are often vulnerable groups and indicate they are unable/unwilling to change.<br>**Overall assessment:** Ghana's food system could potentially adapt to loss of wild meat overall; however severe impacts would be felt by some sectors of society. In rural settings, both consumption and reliance on wild meat for income is greatest, and these communities are the least able to adapt to shocks. An economic shock may be the biggest risk, especially in light of the well-developed commercial trade in wild meat. Female traders and wholesalers who often derive their entire income and livelihood from wild meat are likely to be most affected.	S15-S23-26
**USA**	**Current consumption**: Large absolute volumes of wildmeat consumption. 13.7 million Americans participate in hunting, with food-motivated hunting particularly high in rural areas, driven by preferences for wild meat and limited access to/high prices for commercial meat<br>**Environmental factors**: Agricultural systems are high-yielding and adaptive - could expand or adapt in some areas, though may lead to biodiversity losses in-country, or displacement effects on other countries if cheaper products are imported<br>**Socio-economic factors**: On average, Americans are not lacking protein, however reliance on wild meat and availability of alternatives is heterogenous. Wild meat consumption is higher in rural areas and socially-marginalised communities.<br>**Overall assessment**: Removal of wild meat would mainly impact rural and relatively food-insecure groups. Agricultural expansion may occur, and would need to target rural areas, with a focus on improved supply chains. The hunting industry – and revenues generated for conservation – would suffer large economic losses. Recreational hunters and those with taste preferences and strong attachments to hunting would suffer social costs, and may not comply with prohibitions. Continued sustainable hunting would likely be more beneficial overall.	S27
**China**	**Current consumption:** Estimated that ~12% of total population consume wildlife, though as high as 60% in some regions (e.g. SW China). However, a recent survey shows that >90% of people are against consumption and trade of wildlife following COVID-19.	S28-30

	**Environmental factors:** Agriculture is high-yielding in some areas, though large population and rapidly growing demand means domestic livestock production cannot meet current demand - imports of livestock and feed are increasing, with displacement effects on other countries<br>**Socio-economic factors**: Wild meat is typically consumed for taste, rarity, nourishment and social purposes. Over 14 million people directly employed in wildlife farming industry, which forms a key part of the rural economy, and was once encouraged by the government as part of the poverty alleviation measures. Of these, around 6.3 million people are employed in wildlife farming for human consumption. However, >90% of educated urban people support more stringent regulation of wildlife consumption and trade following COVID-19.<br>**Overall assessment:** China's food system could potentially adapt to loss of wild meat overall, though increases in agricultural production (or imports from elsewhere) will be required, with risks to biodiversity and EIDs. However, given the role of wildlife farming in providing employment for rural people, there could be significant economic shocks in rural areas. If farms are closed without rapid investment in new economic activities or shifting of eating habits, people may turn to illegal hunting and trading of wild species, and/or agriculture, with implications for biodiversity loss. Continued faming of low-risk species (e.g. reptiles, amphibians) would likely be more beneficial overall.	
**Nigeria**	**Current consumption:** Communities in close proximity to wildlife regularly hunt, process and/or consume wild meat (e.g. >99% of people hunt and consume around Cross River National Park, >52% of people hunt and sell around Old Oyo National Park, 11% hunt in Otukpo)<br>**Environmental factors:** Well-developed, high-value agriculture sector in Nigeria, which could be expanded or intensified. Extremely high rates of tropical deforestation, driven to a large degree by agricultural expansion, and continued pressure on remaining tropical forest, which is both a hotspot of mammal biodiversity and EID risk. Seasonality strongly influences hunting – preferable conditions in dry season.<br>**Socio-economic factors**: Wild meat is used for food, income, taste and cultural reasons. Studies show preferences for wild meat over domestic meat. However, hunting is considered an undesirable livelihood – it is challenging, people are aware of zoonotic disease risks, and indicate willingness to change with provision of alternatives. Evidence that Ebola-related campaign discouraged wild meat consumption.<br>**Overall assessment:** Nigeria's food system could potentially adapt to loss of wild meat through expansion of animal agriculture and provision of alternatives to rural communities, though with concomitant risks for biodiversity and EIDs. Taste preferences for wild meat over domestic meat would remain a challenge, though public health messaging may overcome this. Alternative protein sources that satisfy taste preferences – such as small-scale wildlife farming or sustainably managed hunting of low-disease-risk species – may be more effective.	S31-33

Table S3. Detailed information on case study places. Related to Table 1.

Supplemental references

S1. Merson, S.D., Dollar, L.J., Johnson, P.J., and Macdonald, D.W. (2019). Poverty not taste drives the consumption of protected species in Madagascar. Biodivers Conserv *28*, 3669–3689.

S2. Borgerson, C., Randrianasolo, J.F., Andraina, T.R., Anjaranirina, E.J.G., Randriamady, H.J., Merson, S., Dollar, L., and Golden, C.D. (2020). Wildlife hunting in complex human-environmental systems: How understanding natural resource use and human welfare can improve conservation in the Ankarafantsika National Park, Madagascar. Madagascar Conserv Dev *14*, 37–45.

S3. Poudyal, M., Jones, J.P.G., Rakotonarivo, O.S., Hockley, N., Gibbons, J.M., Mandimbiniaina, R., Rasoamanana, A., Andrianantenaina, N.S., and Ramamonjisoa, B.S. (2018). Who bears the cost of forest conservation? PeerJ *2018*, e5106.

S4. Jenkins, R.K.B., Keane, A., Rakotoarivelo, A.R., Rakotomboavonjy, V., Randrianandrianina, F.H., Razafimanahaka, H.J., Ralaiarimalala, S.R., and Jones, J.P.G. (2011). Analysis of patterns of bushmeat consumption reveals extensive exploitation of protected species in eastern madagascar. PLoS One *6*, 1–11.

S5. Koppert, G.J., Dounias, E., Froment, A., and Pasquet, P. (1996). Food consumption in three forest populations of the southern coastal area of Cameroon : Yassa - Mvae - Bakola. In Tropical Forests, People and Food: Biocultural Interactions And Applications to Development, C. Hladik, O. Linares, H. Pagezy, A. Semple, and M. Hadley, eds. (UNESCO), pp. 295–310.

S6. Booker, F. (2019). Why Eat Wild Meat? Results of a literature review on drivers of wild meat as a food choice.

S7. Kleinschroth, F., Laporte, N., Laurance, W.F., Goetz, S.J., and Ghazoul, J. (2019). Road expansion and persistence in forests of the Congo Basin. Nat Sustain *2*, 628–634.

S8. van Velden, J.L., Travers, H., Moyo, B.H.Z., and Biggs, D. (2020). Using scenarios to understand community-based interventions for bushmeat hunting and consumption in African savannas. Biol Conserv *248*, 108676.

S9. Maseko, H., Shackleton, C.M., Nagoli, J., and Pullanikkatil, D. (2017). Children and Wild Foods in the Context of Deforestation in Rural Malawi. Hum Ecol *45*, 795–807.

S10. van Velden, J.L., Wilson, K., Lindsey, P.A., McCallum, H., Moyo, B.H.Z., and Biggs, D. (2020). Bushmeat hunting and consumption is a pervasive issue in African savannahs: insights from four protected areas in Malawi. Biodivers Conserv *29*, 1443–1464.

S11. Wilkie, D.S., Starkey, M., Abernethy, K., Effa, E.N., Telfer, P., and Godoy, R. (2005). Role of prices and wealth in consumer demand for bushmeat in Gabon, Central Africa. Conserv Biol *19*, 268–274.

S12. van Vliet, N., and Nasi, R. (2008). Hunting for livelihood in Northeast Gabon: Patterns, evolution, sustainability. Ecol Soc *13*.

S13. Abernethy, K., and Ndong Obiang, A.M. (2010). Bushmeat in Gabon.

S14. Mcnamara, J., Robinson, E.J.Z., Abernethy, K., Midoko, D., Hannah, I., and Juliet, N.K.S. (2020). COVID - 19 , Systemic Crisis , and Possible Implications for the Wild Meat Trade in Sub - Saharan Africa. Environ Resour Econ.

S15. Van Gils, E.J.T., Ingram, V.J., Iponga, D.M., and Abernethy, K. (2019). Changes in Livelihood Practices, Strategies and Dependence on Bushmeat in Two Provinces in Gabon. Int For Rev *21*, 108–127.

S16. Chaves, W.A., Valle, D.R., Monroe, M.C., Wilkie, D.S., Sieving, K.E., and Sadowsky, B. (2018). Changing Wild Meat Consumption: An Experiment in the Central Amazon, Brazil. Conserv Lett *11*, 1–10.

S17. Antunes, A.P., Rebêlo, G.H., Pezzuti, J.C.B., Vieira, M.A.R. de M., Constantino, P. de A.L., Campos-Silva, J.V., Fonseca, R., Durigan, C.C., Ramos, R.M., Amaral, J.V. do, et al. (2019). A conspiracy of silence: Subsistence hunting rights in the Brazilian Amazon. Land use policy *84*, 1–11.

S18. Peres, C.A. (2000). Effects of Subsistence Hunting on Vertebrate Community Structure in Amazonian Forests.

S19. El Bizri, H.R., Morcatty, T.Q., Valsecchi, J., Mayor, P., Ribeiro, J.E.S., Vasconcelos Neto, C.F.A., Oliveira, J.S., Furtado, K.M., Ferreira, U.C., Miranda, C.F.S., et al. (2020). Urban wild meat consumption and trade in central Amazonia. Conserv Biol *34*, 438–448.

S20. Strassburg, B.B.N., Beyer, H.L., Crouzeilles, R., Iribarrem, A., Barros, F., de Siqueira, M.F., Sánchez-Tapia, A., Balmford, A., Sansevero, J.B.B., Brancalion, P.H.S., et al. (2019). Strategic approaches to restoring ecosystems can triple conservation gains and halve costs. Nat Ecol Evol *3*, 62–70.

S21. Castilho, L.C., De Vleeschouwer, K.M., Milner-Gulland, E.J., and Schiavetti, A. (2019). Hunting of

mammal species in protected areas of the southern Bahian Atlantic Forest, Brazil. ORYX *53*, 687–697.
S22. Sousa, J.A.C., and Srbek-Araujo, A.C. (2017). Are we headed towards the defaunation of the last large Atlantic Forest remnants? Poaching activities in one of the largest remnants of the Tabuleiro forests in southeastern Brazil. Environ Monit Assess *189*, 1–13.
S23. Brashares, J.S., Golden, C.D., Weinbaum, K.Z., Barrett, C.B., and Okello, G. V. (2011). Economic and geographic drivers of wildlife consumption in rural Africa. Proc Natl Acad Sci U S A *108*, 13931–13936.
S24. Shanti-Alexander, J., McNamara, J., Rowcliffe, J.M., Oppong, J., and Milner-Gulland, E.J. (2015). The role of bushmeat in a West African agricultural landscape. ORYX *49*, 643–651.
S25. Schulte-Herbrüggen, B., Cowlishaw, G., Homewood, K., and Rowcliffe, J.M. (2013). The Importance of Bushmeat in the Livelihoods of West African Cash-Crop Farmers Living in a Faunally-Depleted Landscape. PLoS One *8*, e72807.
S26. McNamara, J., Rowcliffe, M., Cowlishaw, G., Alexander, J.S., Ntiamoa-Baidu, Y., Brenya, A., and Milner-Gulland, E.J. (2016). Characterising wildlife trade market supply-demand dynamics. PLoS One *11*.
S27. Conservation Visions Inc (2016). Consumption patterns of wild protein in North America: a literature review in support of the wild harvest initiative.
S28. Wang, H., Shao, J., Luo, X., Chuai, Z., Xu, S., Geng, M., and Gao, Z. (2020). Wildlife consumption ban is insufficient. Science (80-) *367*, 1435–1435.
S29. Zhang, L., Hua, N., and Sun, S. (2008). Wildlife trade, consumption and conservation awareness in southwest China. Biodivers Conserv *17*, 1493–1516.
S30. Shi, X., Zhang, X., Xiao, L., Li, B. V, Liu, J., Yang, F., Zhao, X., and Cheng, C. (2020). Public perception of wildlife consumption and trade during the COVID-19 outbreak. Biodivers Sci *28*, 630–643.
S31. Halidu, S.K. (2019). Assessment of bush meat sale and its implication on wildlife conservation in Old Oyo National Park , Nigeria. World News Nat Sci *23*, 266–275.
S32. Friant, S., Paige, S.B., and Goldberg, T.L. (2015). Drivers of Bushmeat Hunting and Perceptions of Zoonoses in Nigerian Hunting Communities. PLoS Negl Trop Dis *9*, 1–16.
S33. Fa, J.E., Seymour, S., Dupain, J., Amin, R., Albrechtsen, L., and Macdonald, D. (2006). Getting to grips with the magnitude of exploitation: Bushmeat in the Cross-Sanaga rivers region, Nigeria and Cameroon. Biol Conserv *129*, 497–510.
