# Supplementary material for: Investigating the risks of removing wild meat from global food systems
Source: Curr Biol. 2021 Apr 26;31(8):1788–1797.e3. doi: 10.1016/j.cub.2021.01.079 (PMC8094154; doi:10.1016/j.cub.2021.01.079)
Supplement: Document S2. Article plus supplemental information [file mmc2.pdf]

Report

Current Biology

Investigating the risks of removing wild meat from global food systems

Graphical Abstract

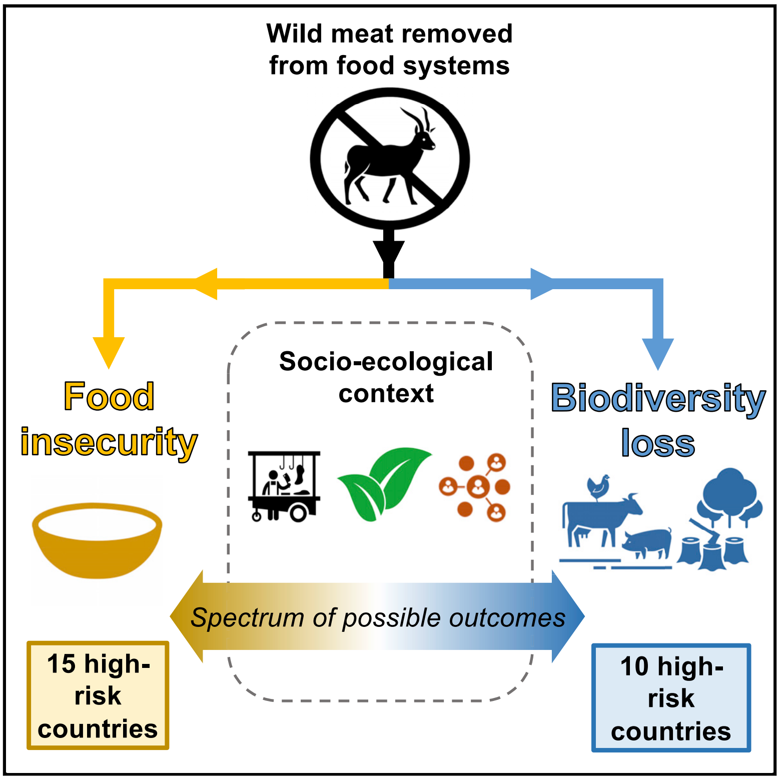

Authors

Hollie Booth, Michael Clark,
E.J. Milner-Gulland, ...,
Cédric Thibaut Kamogne Tagne,
Julia van Velden, David R. Williams

Correspondence

hollie.booth@zoo.ox.ac.uk

In Brief

The COVID-19 pandemic has led to calls to prohibit wild meat consumption, to protect public health and biodiversity. However, Booth et al. demonstrate that the sudden removal of wild meat from food systems could negatively impact people and nature. Wildlife trade policy interventions need to consider tele-couplings between food systems and nature.

Highlights

- 15 countries identified as at risk of food insecurity from wild meat prohibitions
- Extra agricultural land to replace wild meat protein with livestock is ~124,000 km^2
- This land-use change could drive >260 species toward extinction, globally
- Context-specific factors moderate risks of food insecurity and biodiversity loss

Booth et al., 2021, Current Biology *31*, 1788–1797
April 26, 2021 © 2021 The Authors. Published by Elsevier Inc.
https://doi.org/10.1016/j.cub.2021.01.079

CellPress

CellPress
OPEN ACCESS

Current Biology

Report

Investigating the risks of removing wild meat from global food systems

Hollie Booth,[1,18,19,*] Michael Clark,[1,2] E.J. Milner-Gulland,[1] Kofi Amponsah-Mensah,[3] André Pinassi Antunes,[4,5] Stephanie Brittain,[1] Luciana C. Castilho,[6] João Vitor Campos-Silva,[7,8] Pedro de Araujo Lima Constantino,[5] Yuhan Li,[1] Lessah Mandoloma,[9] Lotanna Micah Nneji,[10] Donald Midoko Iponga,[11] Boyson Moyo,[9] James McNamara,[12] O. Sarobidy Rakotonarivo,[13] Jianbin Shi,[14] Cédric Thibaut Kamogne Tagne,[15] Julia van Velden,[16] and David R. Williams[17]

[1]The Interdisciplinary Centre for Conservation Science (ICCS), Department of Zoology, University of Oxford, Oxford, UK
[2]Oxford Martin School and Nuffield Department of Population Health, University of Oxford, Oxford, UK
[3]Centre for African Wetlands, University of Ghana, Legon, Accra, Ghana
[4]Department of Ecology, National Institute of Amazonian Research, Brazil
[5]RedeFauna—Rede de Pesquisa em Diversidade, Conservação e Uso da Fauna da Amazônia, 70879-070, Brasília, DF, Brazil
[6]Ethnoconservation and Protected Areas Laboratory, State University of Santa Cruz, Ilhéus, Bahia, Brazil
[7]Faculty of Ecology and Natural Resource Management, Norwegian University of Life Sciences, 1430 Ås, Norway
[8]Instituto de Ciências Biológicas e da Saúde, Universidade Federal de Alagoas, AL Maceió, Brazil
[9]Lilongwe University of Agriculture and Natural Resource, Malawi
[10]Kunming Institute of Zoology, Chinese Academy of Sciences, PR China
[11]Institut de recherche en ecologie tropicale, Centre National de la Recherche Scientifique et Technologique, Libreville, Gabon
[12]Conservation Research Consultants, London, UK
[13]Ecole Supérieure des Sciences Agronomiques, Université d'Antananarivo, Madagascar
[14]School of Environment, Beijing Normal University Beijing 100875, China
[15]Fondation Camerounaise Terre Vivante, Cameroon
[16]Environmental Futures research institute, Griffith University, Australia
[17]Sustainability Research Institute, School of Earth and Environment, University of Leeds, Leeds, UK
[18]Twitter: @ICCS_updates
[19]Lead contact
*Correspondence: hollie.booth@zoo.ox.ac.uk
https://doi.org/10.1016/j.cub.2021.01.079

SUMMARY

The COVID-19 pandemic has brought humanity's strained relationship with nature into sharp focus, with calls for cessation of wild meat trade and consumption, to protect public health and biodiversity.[1,2] However, the importance of wild meat for human nutrition, and its tele-couplings to other food production systems, mean that the complete removal of wild meat from diets and markets would represent a shock to global food systems.[3–6] The negative consequences of this shock deserve consideration in policy responses to COVID-19. We demonstrate that the sudden policy-induced loss of wild meat from food systems could have negative consequences for people and nature. Loss of wild meat from diets could lead to food insecurity, due to reduced protein and nutrition, and/or drive land-use change to replace lost nutrients with animal agriculture, which could increase biodiversity loss and emerging infectious disease risk. We estimate the magnitude of these consequences for 83 countries, and qualitatively explore how prohibitions might play out in 10 case study places. Results indicate that risks are greatest for food-insecure developing nations, where feasible, sustainable, and socially desirable wild meat alternatives are limited. Some developed nations would also face shocks, and while high-capacity food systems could more easily adapt, certain places and people would be disproportionately impacted. We urge decision-makers to consider potential unintended consequences of policy-induced shocks amidst COVID-19; and take holistic approach to wildlife trade interventions, which acknowledge the interconnectivity of global food systems and nature, and include safeguards for vulnerable people.

RESULTS

A global perspective on the potential negative consequences of removing wild meat from food systems

To investigate the potential negative consequences of the sudden policy-induced loss of wild meat from food systems (e.g., due to prohibitions on wild meat trade and consumption in response to COVID-19), we explored global patterns in two contrasting 'worst-case scenarios'. A worst-case scenario for food insecurity is one in which all wild meat is suddenly removed from food systems, in the absence of feasible, socially desirable alternatives, such that the lost protein and nutrients are not

 © 2021 The Authors. Published by Elsevier Inc.
This is an open access article under the CC BY license (http://creativecommons.org/licenses/by/4.0/).

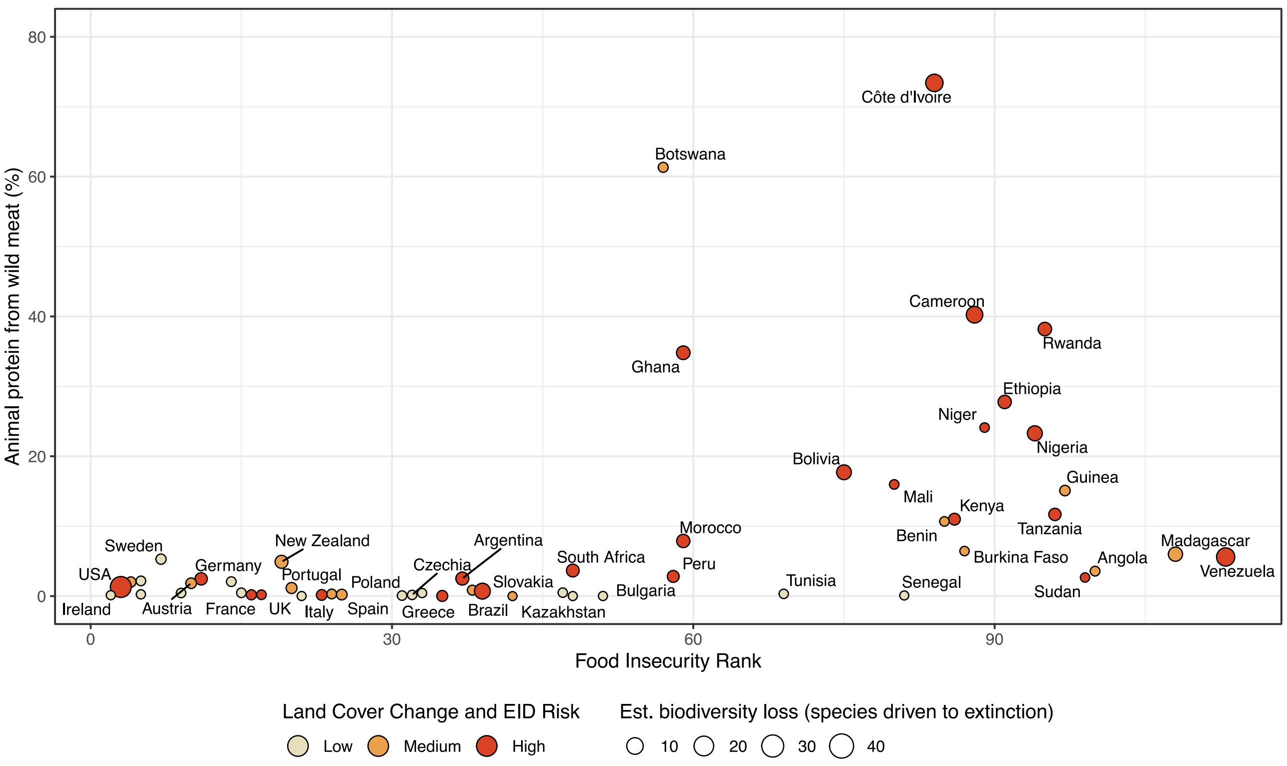

Figure 1. Summarizing global patterns in the risk of negative consequences of bans on wildlife trade and consumption for 54 countries

Countries at high risk of food insecurity are located in the top right-hand corner (e.g., Côte D'Ivoire and Botswana) and extreme right of the figure (e.g., Madagascar, where per capita protein intake could fall below minimum healthy intake, as recommended by the World Health Organization; as per Figure S1). Countries at highest risk of land use change, biodiversity loss and elevated EID risk are larger red circles. Countries which are both in the top right hand-corner and have larger red circles could face the severest trade-offs between lost protein, or land-use change and a loss of biodiversity to replace the protein. See Tables S1 and S2 for data, and STAR methods for data sources. N.B. Several countries known to have high wild meat consumption (e.g., Sierra Leone, Gabon, DR Congo, Uganda) are not included here due to lack of data, while no food insecurity rank was available for Republic of Congo, Zimbabwe and Central African Republic.

replaced. Conversely, if all wild meat is replaced by animal agriculture, this could lead to a worst-case land-use change scenario, with subsequent impacts on biodiversity loss and the risk of emerging infectious diseases (EIDs). High-quality data on wild meat consumption at a global scale is limited. However, by drawing together available global datasets on nutrient supply and land demand for biodiversity[4,7–11] we provide a rudimentary estimate of the animal protein that would be lost from diets if all wild meat consumption ceased, and the land required to replace this protein with livestock production, for 83 countries.

Food insecurity

The sudden loss of wild meat from national food systems, and the ability of countries' food systems to absorb these shocks, are unequally distributed, with risks of protein shortfalls in some of the world's most food-insecure countries. We identified 15 countries at high risk of food insecurity, which rely on wild meat for more than 5% of total animal protein, and are currently ranked in the bottom 50% of the global food security index (Figure 1; Table S1). Overall, Côte d'Ivoire and Botswana were identified as having the highest reliance on wild meat, deriving 73% and 61% of animal protein from wild meat, respectively, and ranking 84th and 57th (out of 113) for global food insecurity, respectively. Eight countries could be at especially high risk of protein deficiencies, because loss of wild meat without immediate replacement could cause mean per capita protein supplies to fall below World Health Organization (WHO) recommended minimum intakes. These countries, all of which are in Sub-Saharan Africa, are: Madagascar, Republic of Congo, Guinea, Rwanda, Central African Republic, Zimbabwe, Botswana, Côte d'Ivoire (Figure S1). Prohibitions on wildlife use could exacerbate existing food insecurity in these countries, especially if implemented without rapid provision of alternatives. However, wild meat consumption is not limited to food-insecure countries: 10 countries which are members of the Organization for Economic Co-operation and Development (OECD), and therefore have high-income economies/very high Human Development Indexes, source at least 1% of protein from wild meat. These countries are: Austria, Colombia, Denmark, Germany, New Zealand, Norway, Portugal, Switzerland, Sweden, and the USA, with the USA being the world's third largest reported wild meat consumer in absolute terms (53.6 million kg per year), only superseded by Nigeria and Côte d'Ivoire (62.2 and 58.8 million kg per year, respectively) (Figure 1; Table S1). However, low levels of food insecurity/higher food system resilience suggest these countries' food systems could more easily adapt to loss of wild meat (Figure 1).

Land-use change and biodiversity loss

We estimated that 123,980 km^2 of additional agricultural land would be needed to replace wild meat protein with protein from domestic livestock (based on region- and livestock-specific estimates of land demand per unit livestock production, and current livestock consumption) (Table S2). We identified two countries where estimated demand for new agricultural land was over 10,000 km^2: Nigeria (10,320 km^2) and USA (12,282 km^2); and a further seven with 5 - 10,000 km^2: Brazil, Colombia, Ethiopia, Ecuador, Côte d'Ivoire, Bolivia, and Venezuela (Table S2).

Based on country-specific estimates of the species extinction risks associated with this land use change (i.e., the number of species destined to be set on a track toward extinction from agricultural land and land-use change), we estimate that up to 267 species could be driven toward extinction globally, with wide variation in potential biodiversity impacts across countries (Table S2). For many countries, extinction estimates are low (i.e., less than one extinction), however, in the top 10 extinction-estimate countries, at least five species are destined for extinction, with some as high as 40-80 species per country. These top 10 countries are primarily located in South America (Ecuador [85.1 species destined for extinction], Colombia [41.8 species], Venezuela [15.1 species], Brazil [8.2], Bolivia [5.9], and Suriname [6.2]) and Sub-Saharan Africa (Côte d'Ivoire [12.4 species destined toward extinction], Cameroon [10 species], and Nigeria [6.3 species]) as well as the USA (with the third highest number of estimated extinctions, globally: 24.8 species).

Area and rate of increase of pasture and cropland, and absolute livestock and poultry numbers, are also significant predictors of emerging infectious disease (EID) occurrence.[10] As such, rapid increases in land area for animal agriculture may bring elevated EID risk (Figure 1). These risks are further exacerbated in forest regions with high mammalian biodiversity—a classification which includes many of the countries with the highest estimated land demands of replacing wild caught meat.[10,12]

Case studies

In reality, the impacts of prohibitions on wild meat consumption would be moderated by context-specific factors. Acknowledging this, we qualitatively analyzed 10 case studies across a range of contexts, to explore likely outcomes in different places, under different ecological and socio-economic conditions. The cases that may find it most difficult to adapt are represented by Madagascar, rural Gabon, the East Region of Cameroon, Malawi, and the Brazilian Amazon. In these places, wild meat consumption forms an important component of people's diets, and substitutes are not readily available for a range of environmental and socio-economic reasons[13–18] (Table 1, Table S3). However, the lack of viable alternatives, combined with epistemic dissonance, social illegitimacy due to food security trade-offs, and limited enforcement capacity suggest that non-compliance with prohibitions is also likely, such that wild meat consumption may continue illicitly[19] (Table 1, Table S3). Efforts to reduce wild meat consumption will likely require the identification and gradual introduction of alternative protein and nutrient sources in these areas, using participatory approaches to ensure their legitimacy and uptake.[14]

In other places, however, food systems could more easily absorb or adapt to the removal of wild meat. These include places where agriculture is already high-yielding, where there are available land and favorable biotic conditions for agricultural expansion, and/or where food systems are already more diversified, and people have the capacity and willingness to adapt (e.g., China, USA, Nigeria, the Brazilian Atlantic Forest, and tropical south west Ghana, Table 1). However, where animal agriculture represents a likely replacement for wild meat, this would be associated with negative consequences for biodiversity and EID risk. For example: the continued loss and fragmentation of the Atlantic forest in Brazil, which will likely result in extinction of endemic species;[27] and further outbreaks of swine flu, which is already devastating farmers in Nigeria[39] and may be mutating into new strains with pandemic potential in China.[40] In addition, if rapidly growing demand for commercial meat cannot be met by domestic agriculture (e.g., in China), imports may increase,[41,42] thus displacing biodiversity and EID risks elsewhere. Importantly, while these food systems may be more adaptable on average, the impacts and adaptive burden would be heterogenous across groups and households, and other economic, social and cultural costs may be significant. For example, rural wildlife farmers in China and female traders in Ghana could suffer major economic shocks if wildlife markets closed, while the rights and cultural values of indigenous populations in the Brazilian Atlantic forest (and indigenous territories throughout the world) would be violated if all hunting and consumption were prohibited (Table 1, Table S3). Such groups are already vulnerable to food-system shocks, and closing wildlife markets may remove an important socio-economic and nutritional safety net. Even in countries with high-yielding food systems, like the USA, access to other forms of animal protein and nutrients would need to expand for rural and marginalised communities that are relatively more dependent on wildlife.[32] The social costs for recreational hunters in the USA, and the economic cost to conservation organizations that rely on hunting permits for income, would also be significant and difficult to replace. The contrasting outlooks for two regions in Brazil (the tropical Amazon and the Atlantic Forest) highlights the heterogeneity of wildlife use within countries, demonstrating how the resilience and adaptability of food systems vary with socioeconomic and biological context, cultural practices and landscape features and enforcement dissonance (Table 1, Table S3). All of these factors should be considered when designing policy interventions in wildlife markets.

DISCUSSION

Calls for prohibitions on wildlife use and trade are motivated by the desire to protect public health and biodiversity. However, our analyses reveal that overly stringent policies risk negative consequences for food security, biodiversity and public health, due to displacement and trade-offs within the broader food system. Appropriate policy formulation must consider equity issues and the rights of indigenous and tribal peoples; be informed by place-specific understandings of food systems and their adaptive capacities; and weigh-up the entire range of costs and benefits of different policy scenarios, including potential displacement of food system impacts.

Acknowledging inequity

As our results show, some of the world's least developed countries (e.g., Côte d'Ivoire, Madagascar, Republic of Congo;

Table 1. Summary of descriptive case studies for 10 places

		Resilience and adaptability			
Case study	Current consumption/ dependence on wild meat	Eco-logical	Socio-economic	Overall outlook	Key refs
**Madagascar**	Ubiquitous and very high	Very Low	Very Low	Food system would struggle to adapt; protein intake may fall leading to malnutrition. Prohibitions may be socially illegitimate and difficult to enforce.	18,20
**East Region, Cameroon**	Ubiquitous and high	Low	Low	Rural food system would struggle to adapt. Prohibitions may be socially illegitimate and difficult to enforce.	16,21
**Malawi**	Moderate, dependence varies in urban versus rural	Low	Low	Rural food system would struggle to adapt, additional prohibitions may be socially illegitimate, with persistence of informal markets. Urban Malawians consuming wild meat (mice and birds) as delicacies may adapt.	17,22
**Rural Gabon**	Ubiquitous and high	Low	Very Low	Rural food system would struggle to adapt. Urbanisation reduces hunting, though demand may remain due to increased wealth and preferences. Prohibitions may be socially illegitimate and difficult to enforce, even with alternatives.	23–25
**Brazilian Amazon**	Ubiquitous and high	High	Very Low	Rural and indigenous food system would struggle to adapt. Reliance on fishing may increase, agricultural expansion may occur to supply urban consumers. High social costs for rural and indigenous peoples, prohibitions difficult to enforce.	13,26
**Brazilian Atlantic Forest**	Moderate	Moderate	Moderate	Food system could potentially adapt; though agricultural expansion should focus on intensification of production and recovery of degraded areas to avoid further deforestation and threats to biodiversity. Social costs would be high for rural poor and indigenous populations. Current prohibitions are already difficult to enforce.	27–29
**Tropical SW Ghana**	Moderate	Moderate	Moderate	Food system could potentially adapt overall; however severe impacts would be felt by some. Economic shocks may be the biggest risk, for female traders/ wholesalers.	24,30,31
**USA**	Low overall, relatively high in some areas	High	High	Food system can adapt overall; though impacts would be felt by some rural and relatively food-insecure groups. Agricultural expansion may occur, the hunting industry – and revenues generated for conservation – would suffer large economic losses. Social cost for recreational hunters would be high.	32
**China**	Moderate overall, high in some areas	Moderate	High	Food system can adapt overall, though increases in agricultural production or imports would be needed, with risks for biodiversity and EIDs. Significant economic shocks for rural wildlife farmers.	33–35
**Nigeria**	High in rural areas	High	Moderate	Food system could potentially adapt through expansion of animal agriculture and provision of alternatives to rural communities, though with concomitant risks for biodiversity and EIDs. Taste preferences for wild meat over domestic meat would remain challenging, though public health messaging may overcome this.	36–38

Shading corresponds to type of negative consequences that are more likely, as per the spectrum in the conceptual model (see Methods): food insecurity = yellow, land-use change and biodiversity loss = blue. The categoric measures of ecological and socio-economic resilience and adaptability are semiquantitative, based on expert judgement by the authors. See Table S3 for details.

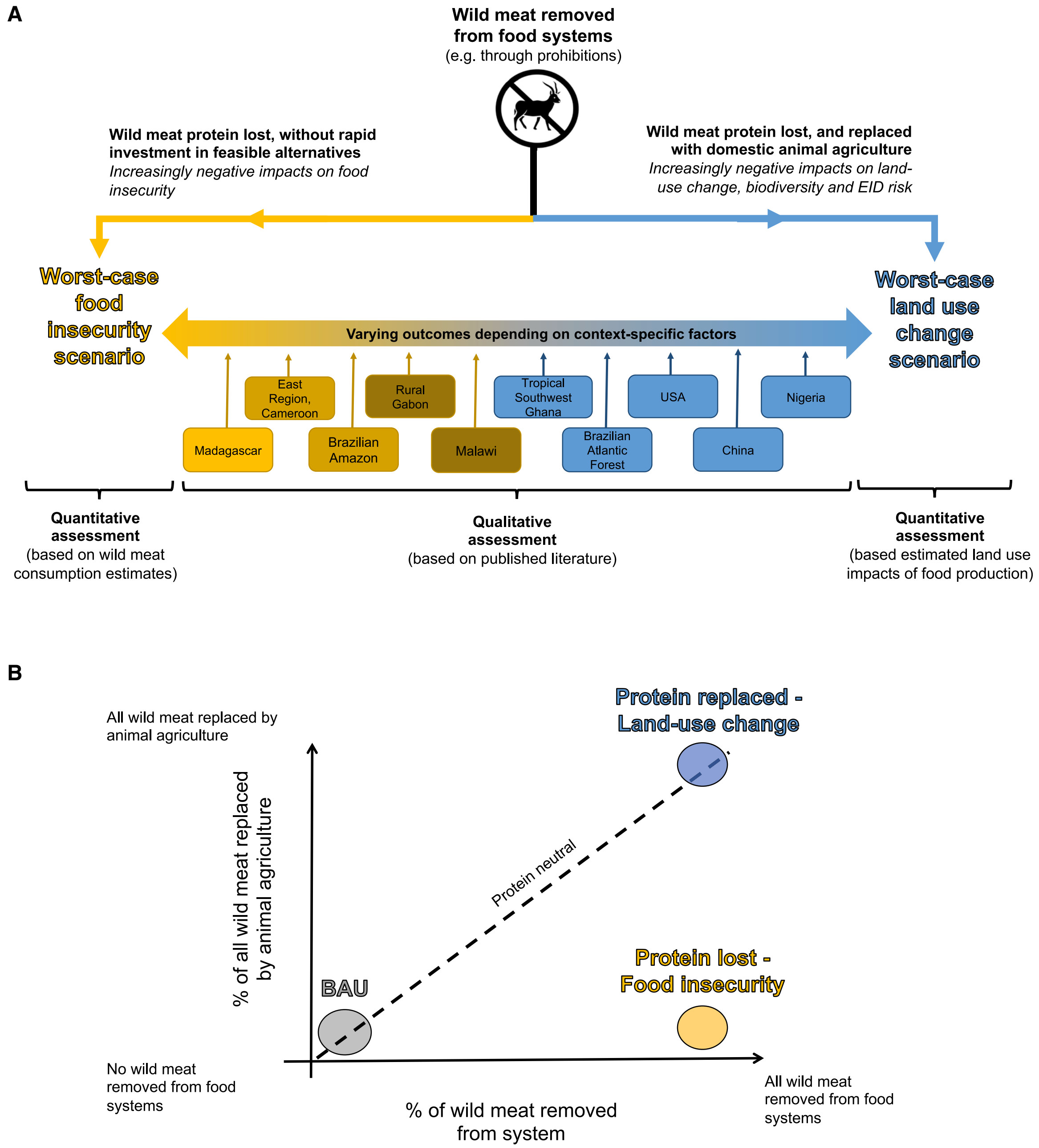

Figure 2. The conceptual framework for this study: a spectrum of negative consequences, and the methods used to assess them

We note that the negative consequences depicted in (A) interact and are inter-dependent, as shown in (B), such that increasing removal of wild meat requires increasing land-use change for animal agriculture in order to maintain current levels of protein. The protein neutral line assumes complete, direct substitution of protein between wild meat sources and animal agriculture source.

Figure 1, Figure S1) are those which are at greatest risk of negative consequences from prohibitions on wild meat. Fragile food systems would struggle to absorb or adapt to loss of wild meat from diets. This could intensify chronic health issues driven by malnutrition, such as stunted growth and impaired cognitive function, with further burdens on society,[43–46] or create severe trade-offs between food security and conservation (Figure 1). These consequences render complete prohibitions impractical or unacceptable in many countries: prohibitions could do more harm than good and raise serious ethical questions regarding the structural inequalities of global wildlife protection.[47]

Importantly, negative consequences would not be uniform within nations (Table 1). Indigenous, rural and socially marginalized groups may be most severely impacted, which could create and accentuate inequalities.[32,48,49] Even in food-secure developed nations like the USA and Canada, which in principle can absorb or adapt to a shock, some marginalized groups, such as migrant and seasonal workers and rural communities, would

Table 2. Summary of all calculations used in quantitative assessment of impacts on food security and land use

Equation 1. Current levels of wild meat consumption

Total annual wild meat consumption per country per annum (**W**)	=	**Equation**	Daily protein (g) per person per day from game meat (W_{PPPD})	X	National population estimate	X	365.25
		**Data source**	GENuS database[7]		UN 2019 population estimates[82]		Days per year

Equation 2. Hypothetical protein consumption if under worst-case food insecurity scenario

Hypothetical protein deficit if wild meat is removed without alternatives (**$P_{removal}$**)	=	**Equation**	Total protein intake per person per day from all foods ($P_{current}$)	-	W_{PPPD}
		**Data source**	GENuS database (Smith, 2016)		GENuS database[7]

Equation 3. Hypothetical land demand under worst-case land use change scenario

Hypothetical land use change (km^2) if all wild meat protein is replaced with animal agriculture (**L_{demand}**)	=	**Equation**	W	X	$\left(\underbrace{\sum_{i-1}^{n} Lpas}_{\text{Pasture demand per unit of meat replacement}} + \underbrace{\sum_{i-1}^{n} Lcrop_i}_{\text{Cropland demand per unit of meat replacement}} \right)$
		**Data source**	Equation 1		Where *i* is the different livestock sectors within a country (beef, sheep/goat, pork, poultry), weighted according to current consumption levels (estimated from Smith (2016)), and L is land needed per sector (km^2/kg) based on region-specific estimates of land demand per unit of protein, for pasture (Lpast) and cropland for feed (Lcrop)[11]

Equation 4. Hypothetical biodiversity loss under worst-case land use change scenario

Hypothetical biodiversity loss (no. species) if all wild meat protein is replaced with animal agriculture (**B_{loss}**)	=	**Equation**	$(L_{demand}(\text{pasture}) \;\text{X}\; (Cpast + (10 \text{ x } Opast))) + (L_{demand}(\text{crop}) \;\text{X}\; (Ccrop + (10 \text{ x } Opast)))$
		**Data**	Where L_{demand} (pasture) is the land demand component for pasture only and L_{demand} (Crop) is the land demand component for crop only (Equation 3). **C** and **O** are country-specific "global characterization factors" of the number of species destined for extinction, caused by: **C** = one-off impact of conversion of natural habitats to pasture (**C**past) and cropland (**C**crop). **O** = ongoing impact of land occupation (assumed to be 10 years in this study) by pasture (**O**past) and cropland (**O**crop)[8]

be impacted nutritionally, economically and culturally.[32,50] In contrast, some groups, such as wealthy urban populations who consume wildlife as a luxury good,[32,48] may find it easier to adapt. Additional inequities—beyond the food systems impacts we explore here—include the loss of livelihoods, rights and social values, which may also undermine incentives for sustainable use.[30,32,33,51–53]

Risk-based regulation could be a more practical and socially just approach: preventing the use and trade of slowly reproducing, endangered species, or those with high zoonotic potential (e.g., great apes and bats)[54] while permitting use and trade of faster-growing species with high potential for sustainable management and minimal public health risks (e.g., cane rats, some amphibians, and reptiles).[55] For example, in Amazonia, there are instances of well-regulated subsistence hunting that support biodiversity conservation and human well-being[13] and provide cost-effective strategies to control zoonoses by empowering households and communities to assume responsibility for disease control.[56] In rural Nigeria and China, small-scale farming of low-disease-risk species such as reptiles, amphibians, and cane rats could provide sustainable protein sources, which satisfy local taste preferences, and have lower biodiversity loss and EID risks than conventional domestic livestock.[36,57–59] In some cases, it may be feasible to substitute wild meat with other forms of plant or animal protein; however, such efforts must be sustainable, respect the customs and capacities of affected people, and avoid further habitat degradation and EID risks through expanding human-wildlife-livestock interfaces.[10,14,60] Affected communities should also be included in decision-making, for practical, ethical, and legal reasons.[19,61]

A food systems approach

Risk-based regulation of wildlife use and trade would benefit from better data on wild meat consumption patterns, and the feasibility of substitutes. For example, more than 100 countries were not included in this study due to missing data. Notable omissions include Sierra Leone, Gabon, DR Congo and Uganda, which have been identified as wild meat consumption hotspots in previous local-scale studies.[23,62] The data also include some notable anomalies. For example, Russia has a long history of recreational and food-motivated hunting,[63,64] yet has very low reported domestic consumption (320 kg) in FAO food balance sheets (Figure 1; Table S1). Similarly, several countries in South East Asia (e.g., Indonesia and Malaysia) have large, widespread wildlife markets,[65] yet have zero "game meat" consumption in the GENuS database and FAO food balance sheet. Finally, even where data is available, it may be far below the "true" consumption, due to widespread informal and unmonitored trade networks. For example, we estimate Brazil's national consumption as 16,250,000 kg per annum (Figure 1; Table S1), yet previous studies have estimated that consumption in Amazonia alone may be five times this mass.[66] These omissions and anomalies likely represent inconsistencies in reporting categories and reporting effort. We acknowledge that the datasets used in this study rely on government reporting, and since wild meat is typically an informal sector, consumption will be under-reported, particularly in less developed countries where monitoring is less stringent (and wild meat is often most important). As such, we likely underestimate the food insecurity and land-use change impacts of removing wild meat from global food systems. Future analyses could benefit from broader geographic and demographic coverage of detailed wildlife use surveys (e.g.,[48]), or methods to correct for monitoring and reporting bias, such as those that have been applied to ivory seizures.[67]

It is also possible that fisheries and aquaculture could substitute for wild meat in some areas;[68] or that increases in yield rather than expansion could help to meet demand for animal agricultural, both of which would buffer any biodiversity impacts of a wild meat ban.[69] However, it's unlikely that these represent viable solutions within the rapid time frame that bans on wild meat consumption could take place. The majority of global fish stocks are fished at or over capacity, while falls in fish catches are already threatening food security in low latitude developing nations—many of which overlap with the high-risk nations identified in this analysis.[70–73] Aquaculture can also have significant environmental and social impacts,[74,75] and few countries currently have the technology, infrastructure and capacity to rapidly and sustainably scale-up aquaculture to replace wild meat where it is most needed.[14] Similarly, while there have been examples of rapid agricultural yield increases at the national level in some countries, these require coordinated investment in agricultural extension, resources, infrastructure and education. Historical trends demonstrate that the norm is for yields to increase linearly,[76] and in many of the countries and regions where the impacts of a wild meat ban are likely to be most severe, these increases are very slow indeed.[69] Cultural uptake will also influence the success of these alternatives, such that a better understanding of the place-specific feasibility of fisheries, aquaculture and rapid yield increases, as more sustainable substitutes for wild meat, are needed to guide future interventions.[77] Undoubtedly, wild-meat consumers in some places will face similar issues with converting to agricultural production/adopting domestic meat, and in the absence of other feasible alternatives, may face nutritional shortfalls, or inability to comply with regulations leading to a business-as-usual scenario.

By highlighting the potential negative consequences of widespread prohibitions of wild meat trade and consumption, we urge decision-makers to adopt a risk-based approach to managing wildlife use in response to COVID-19; one which considers all the costs and benefits of wildlife trade - and proposed regulations - on a case-by-case basis.[55,77] A more holistic approach - implemented via targeted disease mitigation at critical control points throughout all human and animal interactions (including animal agriculture)[78] - could help to reduce the risk of future pandemics and conserve wild biodiversity without such widespread negative consequences. Importantly, due consideration should also be given to the broader macro-economic shocks caused by COVID-19, and how these will influence wildlife markets and food systems.[24] Global food systems may become less resilient due to impacts on supply chains and agricultural production, which may increase reliance on wild meat as a safety net in some areas, and potentially increase the negative consequences of prohibiting its consumption. Policy responses to COVID-19 should be holistic and future-proof, to ensure they support recovery from the current social and economic crisis, and set the world on a pathway to sustainability.

STAR★METHODS

Detailed methods are provided in the online version of this paper and include the following:

- KEY RESOURCES TABLE
- RESOURCE AVAILABILITY
  - Lead contact
  - Materials availability
  - Data and code availability
- EXPERIMENTAL MODEL AND SUBJECT DETAILS
- METHOD DETAILS
  - Conceptual framework
  - Quantitative assessment
  - Food insecurity scenario
  - Land-use change scenario
  - Qualitative assessment
- QUANTIFICATION AND STATISTICAL ANALYSIS

SUPPLEMENTAL INFORMATION

Supplemental information can be found online at https://doi.org/10.1016/j.cub.2021.01.079.

ACKNOWLEDGMENTS

H.B. is grateful to the Oxford-NaturalMotion graduate scholarship for funding her DPhil studies. E.J.M.G., T.K.T., and S.B. acknowledge the UK Government's Darwin Initiative, project 25-015 ("Why eat wild meat"?), E.J.M.G., D.M.I., and J.S. acknowledge the UK Research and Innovation's Global Challenges Research Fund (UKRI GCRF) through the Trade, Development and the Environment Hub project (project number ES/S008160/1). M.C. acknowledges funding from the Wellcome Trust, Our Planet Our Health (Livestock, Environment and People), award number 205212/Z/16/Z. J.V.C.-S. acknowledges the Research Council of Norway for funding his post-doc position (grant no. 295650).

AUTHOR CONTRIBUTIONS

H.B. conceptualized the research. H.B., D.R.W., and M.C. designed the research. H.B., D.R.W., M.C., K.A.-M., A.A., S.B., L.C.C., J.V.C.-S., P.A.L.C., Y.L., L.M., L.M.N., D.M.I., B.M., J.M., O.S.R., J.S., C.T.K.T., and J.v.V. performed research and analyzed data. H.B., D.R.W., and M.C. conducted the quantitative analysis, while H.B., K.A.-M., A.A., S.B., L.C.C., J.V.C.-S., P.A.L.C., Y.L., L.M., L.M.N., D.M.I., B.M., J.M., O.S.R., J.S., C.T.K.T., and J.v.V. conducted the qualitative case study analysis. H.B. led on writing the paper, with substantive inputs from all authors. E.J.M.-G. reviewed early drafts of the analysis and manuscript and provided substantive inputs on the research design and the structure and content of the manuscript.

DECLARATION OF INTERESTS

The authors declare no competing interests. HB is also affiliated with the Wildlife Conservation Society.

Received: September 23, 2020
Revised: November 26, 2020
Accepted: January 22, 2021
Published: February 18, 2021

REFERENCES

1. Yang, N., Liu, P., Li, W., and Zhang, L. (2020). Permanently ban wildlife consumption. Science *367*, 1434.2–141434.
2. End The Trade (2020). End The Trade Petition. https://endthetrade.com/.
3. Cottrell, R.S., Nash, K.L., Halpern, B.S., Remenyi, T.A., Corney, S.P., Fleming, A., Fulton, E.A., Hornborg, S., Johne, A., Watson, R.A., et al. (2019). Food production shocks across land and sea. Nat Sustain *2*, 130–137.
4. Halpern, B.S., Cottrell, R.S., Blanchard, J.L., Bouwman, L., Froehlich, H.E., Gephart, J.A., Sand Jacobsen, N., Kuempel, C.D., McIntyre, P.B., Metian, M., et al. (2019). Opinion: Putting all foods on the same table: Achieving sustainable food systems requires full accounting. Proc. Natl. Acad. Sci. USA *116*, 18152–18156.
5. Friant, S., Ayambem, W.A., Alobi, A.O., Ifebueme, N.M., Otukpa, O.M., Ogar, D.A., Alawa, C.B.I., Goldberg, T.L., Jacka, J.K., and Rothman, J.M. (2020). Eating Bushmeat Improves Food Security in a Biodiversity and Infectious Disease "Hotspot". EcoHealth *17*, 125–138.
6. Roe, D., Dickman, A., Kock, R., Milner-Gulland, E.J., Rihoy, E., and 't Sas-Rolfes, M. (2020). Beyond banning wildlife trade: COVID-19, conservation and development. World Dev. *136*, 105121.
7. Smith, M. (2016). "NutrientsByFood_2011_02protein.csv", Nutrient Supplies by Food and Country (2011). Harvard Dataverse, V3. https://dataverse.harvard.edu/file.xhtml?persistentId=doi:10.7910/DVN/UZW5S3/YQJ7AL.
8. Chaudhary, A., Verones, F., de Baan, L., and Hellweg, S. (2015). Quantifying Land Use Impacts on Biodiversity: Combining Species-Area Models and Vulnerability Indicators. Environ. Sci. Technol. *49*, 9987–9995.
9. The Economist Group (2020). Global Food Security Index (GFSI). https://foodsecurityindex.eiu.com/.
10. Allen, T., Murray, K.A., Zambrana-Torrelio, C., Morse, S.S., Rondinini, C., Di Marco, M., Breit, N., Olival, K.J., and Daszak, P. (2017). Global hotspots and correlates of emerging zoonotic diseases. Nat. Commun. *8*, 1124.
11. Poore, J., and Nemecek, T. (2018). Reducing food's environmental impacts through producers and consumers. Science *360*, 987–992.
12. Jenkins, C.N., Pimm, S.L., and Joppa, L.N. (2013). Global patterns of terrestrial vertebrate diversity and conservation. Proc. Natl. Acad. Sci. USA *110*, E2602–E2610.
13. Antunes, A.P., Rebêlo, G.H., Pezzuti, J.C.B., Vieira, M.A.R. de M., Constantino, P. de A.L., Campos-Silva, J.V., Fonseca, R., Durigan, C.C., Ramos, R.M., do Amaral, J.V., et al. (2019). A conspiracy of silence: Subsistence hunting rights in the Brazilian Amazon. Land Use Policy *84*, 1–11.
14. Wicander, S., and Coad, L. (2015). Learning our Lessons : A Review of Alternative Livelihood Projects in Central Africa (IUCN).
15. Merson, S.D., Dollar, L.J., Johnson, P.J., and Macdonald, D.W. (2019). Poverty not taste drives the consumption of protected species in Madagascar. Biodivers. Conserv. *28*, 3669–3689.
16. Booker, Francesca, and Wilson-Holt, Olivia (2020). Why eat wild meat? Factors affecting the success of alternative protein projects (IIED).
17. Maseko, H., Shackleton, C.M., Nagoli, J., and Pullanikkatil, D. (2017). Children and Wild Foods in the Context of Deforestation in Rural Malawi. Hum. Ecol. Interdiscip. J. *45*, 795–807.
18. Borgerson, C., Randrianasolo, J.F., Andraina, T.R., Anjaranirina, E.J.G., Randriamady, H.J., Merson, S., Dollar, L., and Golden, C.D. (2020). Wildlife hunting in complex human-environmental systems: How understanding natural resource use and human welfare can improve conservation in the Ankarafantsika National Park, Madagascar. Madag. Conserv. Dev. *14*, 37–45.
19. Bonwitt, J., Dawson, M., Kandeh, M., Ansumana, R., Sahr, F., Brown, H., and Kelly, A.H. (2018). Unintended consequences of the 'bushmeat ban' in West Africa during the 2013-2016 Ebola virus disease epidemic. Soc. Sci. Med. *200*, 166–173.
20. Pereira, H.M., and Daily, G.C. (2006). Modeling biodiversity dynamics in countryside landscapes. Ecology *87*, 1877–1885.
21. RStudio Team (2020). RStudio: Integrated Development for R.
22. Booth, H., Clark, M., and Williams, D.R. (2020). ZoologyDave/WildlifeTradeNutrition: Code and data for Investigating the Risks of Removing Wild Meat (Zenodo).

23. Abernethy, K., and Ndong Obiang, A.M. (2010). Bushmeat in Gabon (Ministry of Water and Forests, Gabon), 10.13140/RG.2.2.28730.18881.

24. Mcnamara, J., Robinson, E.J.Z., Abernethy, K., Midoko, D., Hannah, I., and Juliet, N.K.S. (2020). COVID - 19, Systemic Crisis, and Possible Implications for the Wild Meat Trade in Sub - Saharan Africa. Environ Resour Econ.

25. Poudyal, M., Jones, J.P.G., Rakotonarivo, O.S., Hockley, N., Gibbons, J.M., Mandimbiniaina, R., Rasoamanana, A., Andrianantenaina, N.S., and Ramamonjisoa, B.S. (2018). Who bears the cost of forest conservation? PeerJ *6*, e5106.

26. Kleinschroth, F., Laporte, N., Laurance, W.F., Goetz, S.J., and Ghazoul, J. (2019). Road expansion and persistence in forests of the Congo Basin. Nat Sustain *2*, 628–634.

27. Tabarelli, M., Pinto, L.P., Silva, J.M.C., Hirota, M., and Bedê, L. (2005). Challenges and opportunities for biodiversity conservation in the Brazilian Atlantic Forest. Conserv. Biol. *19*, 695–700.

28. van Velden, J.L., Wilson, K., Lindsey, P.A., McCallum, H., Moyo, B.H.Z., and Biggs, D. (2020). Bushmeat hunting and consumption is a pervasive issue in African savannahs: insights from four protected areas in Malawi. Biodivers. Conserv. *29*, 1443–1464.

29. Van Gils, E.J.T., Ingram, V.J., Iponga, D.M., and Abernethy, K. (2019). Changes in Livelihood Practices, Strategies and Dependence on Bushmeat in Two Provinces in Gabon. Int. Rev. *21*, 108–127.

30. Shanti-Alexander, J., McNamara, J., Rowcliffe, J.M., Oppong, J., and Milner-Gulland, E.J. (2015). The role of bushmeat in a West African agricultural landscape. Oryx *49*, 643–651.

31. El Bizri, H.R., Morcatty, T.Q., Valsecchi, J., Mayor, P., Ribeiro, J.E.S., Vasconcelos Neto, C.F.A., Oliveira, J.S., Furtado, K.M., Ferreira, U.C., Miranda, C.F.S., et al. (2020). Urban wild meat consumption and trade in central Amazonia. Conserv. Biol. *34*, 438–448.

32. Conservation Visions Inc (2016). Consumption patterns of wild protein in North America: a literature review in support of the wild harvest initiative. Conservation Visions Report Series (St. John's, Newfoundland, Canada: Conservation Visions).

33. Wang, H., Shao, J., Luo, X., Chuai, Z., Xu, S., Geng, M., and Gao, Z. (2020). Wildlife consumption ban is insufficient. Science *367*, 1435.

34. Castilho, L.C., De Vleeschouwer, K.M., Milner-Gulland, E.J., and Schiavetti, A. (2019). Hunting of mammal species in protected areas of the southern Bahian Atlantic Forest, Brazil. Oryx *53*, 687–697.

35. Sousa, J.A.C., and Srbek-Araujo, A.C. (2017). Are we headed towards the defaunation of the last large Atlantic Forest remnants? Poaching activities in one of the largest remnants of the Tabuleiro forests in southeastern Brazil. Environ. Monit. Assess. *189*, 129.

36. Friant, S., Paige, S.B., and Goldberg, T.L. (2015). Drivers of bushmeat hunting and perceptions of zoonoses in Nigerian hunting communities. PLoS Negl. Trop. Dis. *9*, e0003792.

37. McNamara, J., Rowcliffe, M., Cowlishaw, G., Alexander, J.S., Ntiamoa-Baidu, Y., Brenya, A., and Milner-Gulland, E.J. (2016). Characterising wildlife trade market supply-demand dynamics. PLoS ONE *11*, e0162972.

38. Zhang, L., Hua, N., and Sun, S. (2008). Wildlife trade, consumption and conservation awareness in southwest China. Biodivers. Conserv. *17*, 1493–1516.

39. Omotosho, O. (2020). Why Nigeria's African swine fever outbreak will hit farmers hard. Conversat. https://theconversation.com/why-nigerias-african-swine-fever-outbreak-will-hit-farmers-hard-140764.

40. Sun, H., Xiao, Y., Liu, J., Wang, D., Li, F., Wang, C., Li, C., Zhu, J., Song, J., Sun, H., et al. (2020). Prevalent Eurasian avian-like H1N1 swine influenza virus with 2009 pandemic viral genes facilitating human infection. Proc. Natl. Acad. Sci. USA *117*, 17204–17210.

41. Chen, W. (2019). Chinese consumers ignore calls to eat less beef. China Dialogue. https://chinadialogue.net/en/business/11166-chinese-consumers-ignore-calls-to-eat-less-beef/.

42. Caixin Global (2018). Charts of the Day: China's Growing Meat Consumption. Caixin Glob. https://www.caixinglobal.com/2018-10-12/charts-of-the-day-chinas-growing-meat-consumption-101334433.html.

43. Fa, J.E., Olivero, J., Real, R., Farfán, M.A., Márquez, A.L., Vargas, J.M., Ziegler, S., Wegmann, M., Brown, D., Margetts, B., and Nasi, R. (2015). Disentangling the relative effects of bushmeat availability on human nutrition in central Africa. Sci. Rep. *5*, 8168.

44. Golden, C.D., Fernald, L.C.H., Brashares, J.S., Rasolofoniaina, B.J.R., and Kremen, C. (2011). Benefits of wildlife consumption to child nutrition in a biodiversity hotspot. Proc. Natl. Acad. Sci. USA *108*, 19653–19656.

45. Dufour, D.L., Piperata, B.A., Murrieta, R.S.S., Wilson, W.M., and Williams, D.D. (2016). Amazonian foods and implications for human biology. Ann. Hum. Biol. *43*, 330–348.

46. Neumann, C.G., Bwibo, N.O., Murphy, S.P., Sigman, M., Whaley, S., Allen, L.H., Guthrie, D., Weiss, R.E., and Demment, M.W. (2003). Animal source foods improve dietary quality, micronutrient status, growth and cognitive function in Kenyan school children: background, study design and baseline findings. J. Nutr. *133* (11, Suppl 2), 3941S–3949S.

47. Balmford, A., and Whitten, T. (2003). Who should pay for tropical conservation, and how could the costs be met? Oryx *37*, 238–250.

48. Brashares, J.S., Golden, C.D., Weinbaum, K.Z., Barrett, C.B., and Okello, G.V. (2011). Economic and geographic drivers of wildlife consumption in rural Africa. Proc. Natl. Acad. Sci. USA *108*, 13931–13936.

49. Lee, A. (2020). Chinese frog breeders call for help as wildlife trade ban shuts down business. South China Morning Post. https://www.scmp.com/news/article/3053079/chinese-frog-breeders-call-help-wildlife-trade-ban-shuts-down-business.

50. PROOF (2020). Household Food Insecurity in Canada. Food Insecurity Policy Res. https://proof.utoronto.ca/food-insecurity/.

51. Hurley, K., Brewer, C., and Thornton, G.N. (2015). The role of hunters in conservation, restoration, and management of North American wild sheep. Int. J. Environ. Stud. *72*, 784–796.

52. Parker, K., De Vos, A., Clements, H.S., Biggs, D., and Biggs, R. (2020). Impacts of a trophy hunting ban on private land conservation in South African biodiversity hotspots (Conserv Sci Pract).

53. Campos-Silva, J.V., Peres, C.A., Antunes, A.P., Valsecchi, J., and Pezzuti, J. (2017). Community-based population recovery of overexploited Amazonian wildlife. Perspect Ecol Conserv *15*, 266–270.

54. Han, B.A., Kramer, A.M., and Drake, J.M. (2016). Global Patterns of Zoonotic Disease in Mammals. Trends Parasitol. *32*, 565–577.

55. Booth, H., Arias, M., Brittain, S., Challender, D.W.S., Khanyari, M., Kupier, T., Li, Y., Olmedo, A., Oyanedel, R., Pienkowski, T., et al. (2020). "Saving lives, protecting livelihoods, and safeguarding nature": risk-based wildlife trade policy for sustainable development outcomes post-COVID-19. SocArXiv Prepr.

56. Molyneux, D., Hallaj, Z., Keusch, G.T., McManus, D.P., Ngowi, H., Cleaveland, S., Ramos-Jimenez, P., Gotuzzo, E., Kar, K., Sanchez, A., et al. (2011). Zoonoses and marginalised infectious diseases of poverty: where do we stand? Parasit. Vectors *4*, 106.

57. Jori, F., Mensah, G.A., and Adjanohoun, E. (1995). Grasscutter production: an example of rational exploitation of wildlife. Biodivers. Conserv. *4*, 257–265.

58. Jori, F., Lopez-Béjar, M., and Houben, P. (1998). The biology and use of the African brush-tailed porcupine (Atherurus africanus, Gray, 1842) as a food animal. A review. Biodivers. Conserv. *7*, 1417–1426.

59. Natusch, D., Alexander, G., Van Tri, N., and Aust, P. (2020). Snakes make good food. Banning farms won't help the fight against coronavirus. Conversat. https://theconversation.com/snakes-make-good-food-banning-farms-wont-help-the-fight-against-coronavirus-133075.

60. Wright, J.H., Hill, N.A.O., Roe, D., Rowcliffe, J.M., Kümpel, N.F., Day, M., Booker, F., and Milner-Gulland, E.J. (2016). Reframing the concept of alternative livelihoods. Conserv. Biol. *30*, 7–13.

61. Newing, H., and Perram, A. (2019). What do you know about conservation and human rights? Oryx *53*, 595–596.

62. Fa, J.E., Currie, D., and Meeuwig, J. (2003). Bushmeat and food security in the Congo Basin: linkages between wildlife and people's future. Environ. Conserv. *30*, 71–78.

63. Braden, K. (2014). Illegal recreational hunting in Russia: The role of social norms and elite violators. Eurasian Geogr. Econ. *55*, 457–490.

64. Baskin, L.M. (2000). Reindeer husbandry/hunting in Rus sia in the past, present and future. Polar Res. *19*, 23–29.

65. Milner-Gulland, E.J., Bennett, E.L., Abernethy, K., Bakarr, M., Bennett, E., Bodmer, R., Brashares, J., Cowlishaw, G., Elkan, P., Eves, H., et al. (2003). Wild meat: The bigger picture. Trends Ecol. Evol. *18*, 351–357.

66. Peres, C.A. (2001). Effects of Subsistence Hunting on Vertebrate Community Structure in Amazonian Forests. (Conservation Biology). https://doi.org/10.1046/j.1523-1739.2000.98485.x.

67. Underwood, F.M., Burn, R.W., and Milliken, T. (2013). Dissecting the illegal ivory trade: an analysis of ivory seizures data. PLoS ONE *8*, e76539.

68. Brashares, J.S., Arcese, P., Sam, M.K., Coppolillo, P.B., Sinclair, A.R.E., and Balmford, A. (2004). Bushmeat hunting, wildlife declines, and fish supply in West Africa. Science *306*, 1180–1183.

69. Tilman, D., Clark, M., Williams, D.R., Kimmel, K., Polasky, S., and Packer, C. (2017). Future threats to biodiversity and pathways to their prevention. Nature *546*, 73–81.

70. Golden, C.D., Allison, E.H., Cheung, W.W.L., Dey, M.M., Halpern, B.S., McCauley, D.J., Smith, M., Vaitla, B., Zeller, D., and Myers, S.S. (2016). Nutrition: Fall in fish catch threatens human health. Nature *534*, 317–320.

71. Nasi, R., and Fa, J.E. (2015). The Role of Bushmeat in Food Security and Nutrition. XIV World For Congr. 0–11.

72. Antunes, A.P., Fewster, R.M., Venticinque, E.M., Peres, C.A., Levi, T., Rohe, F., and Shepard, G.H., Jr. (2016). Empty forest or empty rivers? A century of commercial hunting in Amazonia. Sci. Adv. *2*, e1600936.

73. Pauly, D., Watson, R., and Alder, J. (2005). Global trends in world fisheries: impacts on marine ecosystems and food security. Philos. Trans. R. Soc. Lond. B Biol. Sci. *360*, 5–12.

74. Black, K. (2001). Environmental impacts of aquaculture (Sheffield Academic Press).

75. Martinez-Alier, J. (2002). The Environmentalism of the Poor (Cheltenham, UK: Edward Elgar Publishing).

76. Grassini, P., Eskridge, K.M., and Cassman, K.G. (2013). Distinguishing between yield advances and yield plateaus in historical crop production trends. Nat. Commun. *4*, 2918.

77. Brittain, S., Booker, F., Milner-Gulland, E.J., Roe, D., Maddison, N., Mouamfon, M., et al. (2020). Wild meat alternative projects : practical guidance for project design (London, UK: IIED).

78. Petrovan, S., Aldridge, D., Bartlett, H., Bladon, A., Booth, H., Broad, S., Broom, D., Burgess, N., Cleaveland, S., Cunningham, A., et al. (2020). Post COVID-19: a solution scan of options for preventing future zoonotic epidemics. OSF. https://osf.io/5jx3g/.

79. FAO (2019). New Food Balances. Food Agric Organ United Nations. http://www.fao.org/faostat/en/#data/FBS.

80. The Economist Intelligence Unit (2019). Global Food Security Index 2019: Strengthening food systems and the environment through innovation and investment (London, UK: The Economist).

81. Smith, M.R., Micha, R., Golden, C.D., Mozaffarian, D., and Myers, S.S. (2016). Global expanded nutrient supply (GENuS) model: A new method for estimating the global dietary supply of nutrients. PLoS ONE *11*, e0146976.

82. Fournié, G., Guitian, J., Desvaux, S., Cuong, V.C., Dung, H., Pfeiffer, D.U., Mangtani, P., and Ghani, A.C. (2013). Interventions for avian influenza A (H5N1) risk management in live bird market networks. Proc. Natl. Acad. Sci. USA *110*, 9177–9182.

83. Wilkie, D.S., Wieland, M., Boulet, H., Le Bel, S., van Vliet, N., Cornelis, D., BriacWarnon, V., Nasi, R., and Fa, J.E. (2016). Eating and conserving bushmeat in Africa. Afr. J. Ecol. *54*, 402–414.

84. WHO (2007). Protein and amino acid requirements in human nutrition (World Health Organization).

85. United Nations Population Division (2020). Population Databases. Dep Econ Soc Aff Popul. https://www.un.org/en/development/desa/population/publications/database/index.asp.

STAR★METHODS

KEY RESOURCES TABLE

REAGENT or RESOURCE	SOURCE	IDENTIFIER
Deposited Data		
Global Expanded Nutrient Supply (GENuS) database: Nutrient Supplies by Food and Country	[7]	https://doi.org/10.7910/DVN/UZW5S3, Table S1
FAO food balance sheet data	[4,79]	Table S1
The Economist Global Food Security Index (GFSI)	[9,80]	Table S1
Country-specific Characterization Factors for land use impacts on biodiversity	[8]	https://doi.org/10.1021/acs.est.5b02507,
Region- and livestock-specific estimates of land demand per gram of protein based on life-cycle assessments	[11]	https://doi.org/10.1126/science.aaq0216,
All code used for the analysis (deposited in Zenodo)	[22]	https://doi.org/10.5281/zenodo.4005563
National-level wild meat consumption estimates	This paper	Table S1
National-level land demand estimates	This paper	Table S2
National-level biodiversity loss estimates	This paper	Table S2

RESOURCE AVAILABILITY

Lead contact

Requests for further information will be fulfilled by the Lead Contact: Hollie Booth (hollie.booth@zoo.ox.ac.uk).

Materials availability

This study did not generate new, unique reagents.

Data and code availability

The datasets and code used and generated during this study are available in the Supplemental Information and at the following Zenodo repository: https://zenodo.org/record/4415553.

EXPERIMENTAL MODEL AND SUBJECT DETAILS

The primary study subject was national-level nutrient supply from food, which is available for 23 individual nutrients across 225 food categories in the GENuS database.[7,81] This dataset is prepared as per the methods outlined in Smith et al. (2016), and maintained by the University of Harvard Chan School of Public Health. We supplemented gaps in the GENuS database with additional data from FAO food balance sheets and a recently compiled dataset of bushmeat consumption,[4,79] which provide government-reported supplies of food items available for human consumption, along with their caloric value and protein and fat content. This dataset is prepared and maintained by the Food and Agriculture Organization of the United Nations (FAO). Other data used in the analysis include the Global Food Security Index (GFSI), which is a quantitative and qualitative benchmarking model that measures drivers of food security across 113 countries,[80] and is prepared and maintained by The Economist Intelligence Unit; country-specific characterization factors of biodiversity loss from conversion of natural habitats to agricultural land, as calculated and published in Chaudhary et al. (2015);[8] and region- and livestock-specific estimates of land demand per gram of protein based on life-cycle assessments, as calculated and published in Poore and Nemecek (2018).[11]

METHOD DETAILS

Conceptual framework

The potential negative consequences of a policy-induced loss of wild meat from food systems exist on a spectrum between two 'worst-case scenarios' (Figure 2). A worst-case scenario for food insecurity is one in which all wild meat is suddenly lost from food systems, in the absence of feasible, socially desirable alternatives, meaning that the protein is not replaced (Figure 2). Conversely, if all wild meat is replaced by animal agriculture, this could lead to a worst-case land-use change scenario, with

subsequent impacts on biodiversity and EID risk. Alternatively, a lack of enforcement or social acceptance of policies to restrict wild meat supply could result in a business as usual (BAU) scenario, where prohibitions have little effect. Prohibitions can also lead to other perverse consequences, such as proliferation of informal and illicit trade networks, which undermines evidence-based surveillance and disease mitigation, and may increase prices and fuel further corruption and inequity in places where enforcement capacity is weak.[19,82] In reality, consequences would likely fall somewhere in between these three extremes (Figure 2), moderated by levels of compliance and modes of adaptation (e.g., adoption of less-damaging alternatives such as wild-caught fisheries, aquaculture, small-mammal farming, sustainable wildlife hunting or cheap food imports), which in turn depend on system-specific socio-ecological factors, such as culture and biomes.[14,19,83]

We used a mixed-methods approach to explore potential negative consequences along this spectrum. We first use the two contrasting "worst-case" scenarios to quantitively explore global patterns in the potential magnitude of negative consequences. We build on early attempts by Fa et al. (2003) to quantify linkages between wild meat and food security, using recently assembled global datasets on protein supply, food security, land-use change and biodiversity loss from different types of agriculture.[4,7–11] We estimate the animal protein that would be lost from diets, should all wild meat be removed from diets, and the land required to replace this protein with livestock production in 83 countries. We acknowledge that these scenarios are unlikely to occur in full, but use them to highlight which countries could face the largest shocks from the loss of wild meat in food systems. We then qualitatively explore how context-specific idiosyncrasies might plausibly affect the consequences of prohibitions on wildlife trade in 10 case study places, that represent a range and diversity of possible outcomes (Figure 2): Madagascar; East Region Cameroon; the Brazilian Amazon; rural Gabon; Malawi; tropical Southwest Ghana; the Brazilian Atlantic Forest; USA; China and Nigeria.

Quantitative assessment

To assess the impacts of the removal of wild meat from food systems we focused on protein as an indicator for the range of important micro- and macro-nutrients sourced from animal meat.[84] We acknowledge that a range of other important nutrients, vitamins and fatty acids are sourced from animal meat,; however, all of these nutrients will scale in proportion with mass consumed, therefore the overall patterns will be similar. We first estimated annual wild meat consumption for every country for which data were available. We based our estimates on the GENuS database[7] and calculated total annual consumption in a country by multiplying consumption of wild meat protein per person per day as (W_{PPPD}, Table 2; Smith, 2016) by the total population of the country in 2019.[85] We assumed all wild meat was categorised as 'game meat' in the GENuS database, though acknowledge this may underestimate wild meat consumption as it may not capture some types of wildlife consumed for food (e.g., wildfowl, farmed reptiles and amphibians), and reporting biases will vary by country, with underreporting likely in places where wild meat is an informal sector. We supplemented these data with additional data from FAO food balance sheets and a recently compiled dataset of bushmeat consumption.[4] For FAO data we calculated the consumption of 'game meat' as the trade balance (imports minus exports) plus the national annual production. For these datasets we further had to convert live-weight into protein, basing calculations on another recently published dataset.[11] In total, this resulted in 83 countries with non-zero estimates of wild meat consumption (Table S1). We acknowledge that these datasets are imperfect, and likely represent conservative estimates of wild meat consumption: they rely on government reporting, yet wild meat is often traded and consumed within informal and subsistence markets, which are likely to be un-reported, particularly in countries where monitoring is less stringent. Nonetheless, they represent the best-available data for a rudimentary global analysis of this important yet overlooked issue.

Food insecurity scenario

To identify countries where loss of wild meat protein could have negative impacts on food security, we plotted consumption of wild meat protein per person per day (W_{PPPD}, Table 2) against global food insecurity rank[9] for 54 countries with data available for both wild meat consumption and food insecurity. Daily per capita consumption of wild meat protein indicates the magnitude of the shock a country's food system might face if wild meat were suddenly removed, while food insecurity rank provides an indication of how robust each country's food systems currently are. For each country, we also estimated hypothetical per capita protein intake in the absence of wild meat with no alternatives ($P_{\mathbf{removal}}$) (i.e., the worst-case food insecurity scenario) as per Equation 2 (Table 2), and identified countries where $P_{\mathbf{removal}}$ falls below recommended healthy intakes of protein according to the World Health Organization.[84] This indicates which countries may face severe protein deficits, though many countries currently consume in excess of the WHO recommended daily intake of protein, and could feasibly reduce protein intake against current levels without major impacts on nutritional security.

Land-use change scenario

To estimate the worst-case land-use change (L_{demand}), we first estimated the production of domestic livestock (beef, sheep/goat, pork, poultry) required to replace all wild meat protein (W), based on their current share of consumption in the country (Equation 3, Table 2). For example, if meat from poultry, beef, and pigs respectively accounted for 20%, 30%, and 50% of a country's current protein consumption from meat, then 20% of wild meat protein would be replaced by protein from poultry, 30% by protein from beef, and 50% by protein from pigs. We then estimated the additional land needed to support the additional production using region- and livestock-specific estimates of land demand per gram of protein, including both pasture and cropland for feed production.[11] We then summed across livestock species to provide an estimate of the total additional agricultural land that would be required in each country (Equation 3, Table 2). Where region-specific land-demand estimates were lacking, we used global estimates. To investigate

the potential negative consequences of this land-use change on biodiversity (B_{loss}), we used country-specific characterization factors of biodiversity loss from conversion of natural habitats to agricultural land.[8] These characterization factors are reported as the number of species destined to become extinct from agricultural activities in the long-term per unit area. These factors are based on Countryside Species-Area Relationships,[20] and species richness and endemism in different countries, and the affinity of different taxonomic groups for different land uses as calculated by Chaudhary et al.[8] Data limitations mean that characterization factors are limited to four groups of terrestrial vertebrates (amphibians, birds, mammals and reptiles), so we likely under-estimate total extinctions likely to result from land-use change and occupation because we do not include other taxa such as plants or insects in our analysis. Separate estimates have been calculated for cropland or pasture, as well as separate estimates of the one-off biodiversity impact of land-cover change and of the annual biodiversity impact of continued occupation and production on cropland or pasture.[8] For each country, we calculated the total biodiversity impact as the sum of the one-off impact of the land-cover transition (calculated as estimated additional cropland (pasture) multiplied by the characterization factor for conversion into cropland (pasture)) and the on-going impact of land occupation over a 10-year period (calculated as estimated additional cropland/pasture multiplied by the characterization factor for the annual impact of production on cropland (pasture); see also Equation 4, Table 2). This likely represents a conservative estimate of the impacts of land-use change as on-going biodiversity loss is likely to continue for longer (e.g., Hendershot et al., 2020). We also consider the impact this land-use change could have on EID risk, since degree of land-use change is known to be a key predictor of EID events.[10]

Qualitative assessment

These analyses provide plausible bounds for the impacts of a reduction in wild meat consumption, but the actual responses of food systems will be idiosyncratic and shaped by local and national context. To explore how context could shape responses, we outline plausible narratives for how policy-induced removal of wild meat (i.e., prohibitions on wildlife trade and consumption) might impact food systems in 10 case studies. We qualitatively investigate drivers of wild meat consumption and overall food system adaptability, considering current levels of consumption and dependence on wild meat, and environmental and socio-economic factors that influence food system resilience and adaptability, e.g., land availability for agricultural expansion; seasonality of agriculture; technological and human capacity; relative price of and access to alternative protein sources; the degree of urbanization and proximity to wildlife; wealth, cultural preferences and willingness to change consumption patterns; and the perceived legitimacy of regulations.[19,48,83]

QUANTIFICATION AND STATISTICAL ANALYSIS

We conducted all quantitative analysis using RStudio,[21] the code has been made publicly available via Zenodo.[22] We did not conduct any statistical analysis in this study.

Current Biology, Volume *31*

Supplemental Information

Investigating the risks of removing wild meat from global food systems

Hollie Booth, Michael Clark, E.J. Milner-Gulland, Kofi Amponsah-Mensah, André Pinassi Antunes, Stephanie Brittain, Luciana C. Castilho, João Vitor Campos-Silva, Pedro de Araujo Lima Constantino, Yuhan Li, Lessah Mandoloma, Lotanna Micah Nneji, Donald Midoko Iponga, Boyson Moyo, James McNamara, O. Sarobidy Rakotonarivo, Jianbin Shi, Cédric Thibaut Kamogne Tagne, Julia van Velden, and David R. Williams

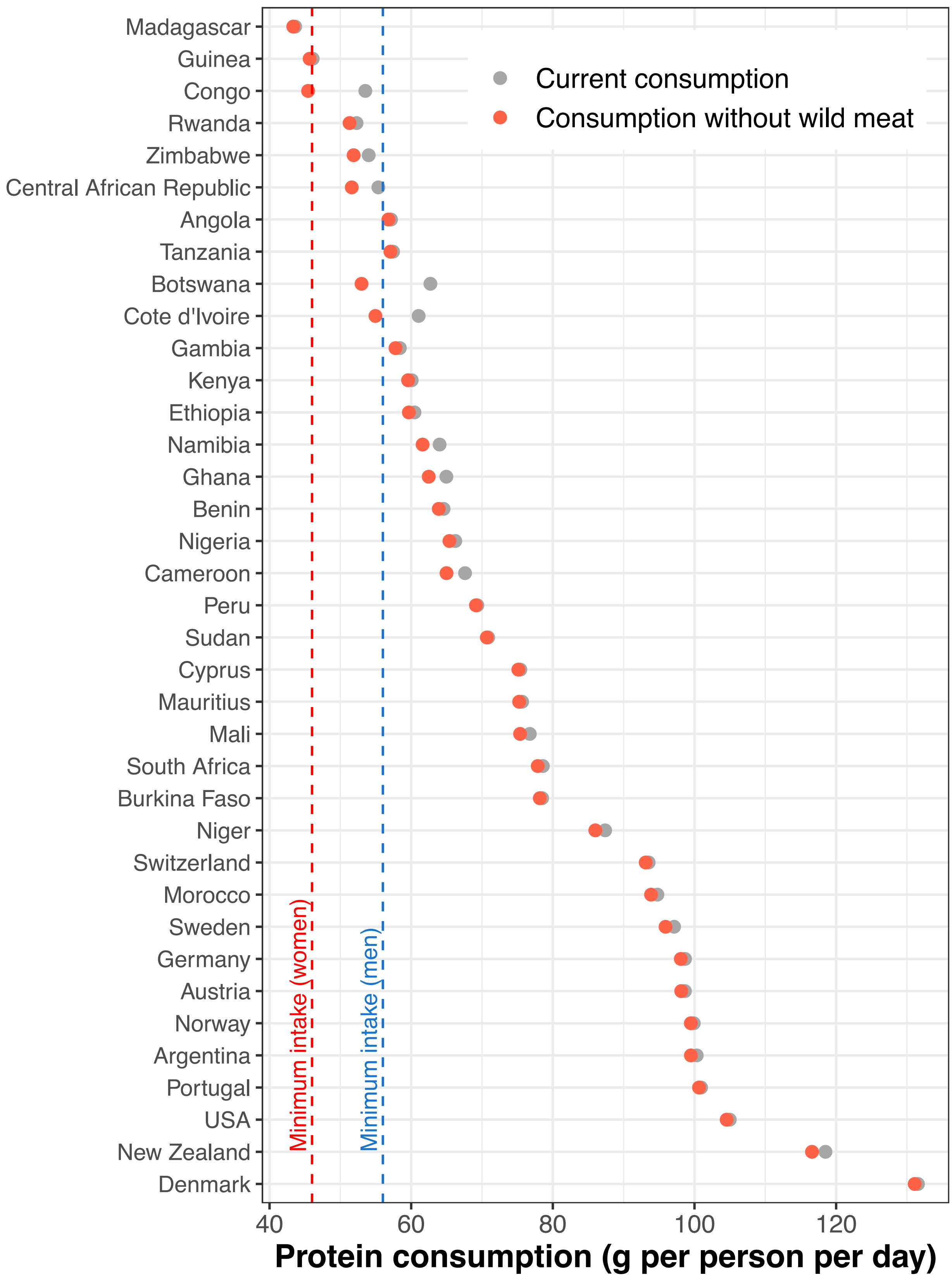

Figure S1. Estimated per capita protein deficits caused by loss of wild meat from diets, in the absence of replacements (current estimated total protein intake minus estimated game meat protein intake). Minimum protein intake values based on guidelines from the World Health Organisation. Relates to Figure 1.

COUNTRY	ISO3	Protein from game meat per person per day - GENUS (g)	Total annual protein from game meat - GENUS (kg)	Protein from all meat per person per day - GENUS (g)	Total annual protein from all meat - GENUS (kg)	Percent protein from game meat	Protein per person per day without game meat (g)	Game meat data source for analysis	Global Food Security Index
Nigeria	NGA	0.83	62,254,085	3.55	267,314,892	23.3	65.40	GENUS	94
Cote d'Ivoire	CIV	6.10	58,766,637	8.31	80,057,453	73.4	54.95	GENUS	84
USA	USA	0.44	53,679,031	34.01	4,111,466,970	1.3	104.54	GENUS	3
Ethiopia	ETH	0.79	33,254,337	2.85	119,751,625	27.8	59.68	GENUS	91
Ghana	GHA	2.49	28,296,302	7.16	81,286,399	34.8	62.48	GENUS	59
Cameroon	CMR	2.62	25,383,805	6.50	63,070,135	40.2	64.99	GENUS	88
Germany	DEU	0.60	18,265,160	24.19	740,156,031	2.5	98.07	GENUS	11
Congo	COG	8.08	16,289,926	17.28	34,827,648	46.8	45.44	GENUS	
South Africa	ZAF	0.72	15,499,301	19.56	423,707,390	3.7	77.87	GENUS	48
Argentina	ARG	0.82	13,602,721	32.83	541,958,029	2.5	99.50	GENUS	37
Niger	NER	1.39	12,264,897	5.75	50,878,149	24.1	86.00	GENUS	89
Morocco	MAR	0.89	12,042,828	11.33	152,739,431	7.9	93.87	GENUS	59
Zimbabwe	ZWE	2.13	11,582,638	7.79	42,302,051	27.4	51.87	GENUS	
Mali	MLI	1.37	10,127,448	8.57	63,418,153	16.0	75.38	GENUS	80
Kenya	KEN	0.52	10,119,492	4.68	91,968,972	11.0	59.57	GENUS	86
Botswa	BWA	9.72	8,352,768	15.86	13,619,480	61.3	52.99	GENUS	57
Tanzania	TZA	0.38	8,232,121	3.23	70,448,317	11.7	57.06	GENUS	96
CAR	CAF	3.76	6,624,268	13.91	24,536,024	27.0	51.61	GENUS	
Rwanda	RWA	0.99	4,667,121	2.58	12,217,856	38.2	51.30	GENUS	95
Sweden	SWE	1.20	4,432,779	22.73	83,839,357	5.3	95.92	GENUS	7
Angola	AGO	0.37	4,409,458	10.29	123,542,743	3.6	56.79	GENUS	100
New Zealand	NZL	1.90	3,347,918	38.78	68,308,487	4.9	116.60	GENUS	19
Iran	IRN	0.11	3,221,848	11.29	346,209,703	0.9	101.57	GENUS	
Benin	BEN	0.68	3,016,751	6.38	28,239,351	10.7	63.89	GENUS	85
Sudan (former)	SDN	0.19	3,001,548	7.04	112,774,175	2.7	70.68	GENUS	99
Madagascar	MDG	0.28	2,797,673	4.61	46,657,906	6.0	43.35	GENUS	108
Burki Faso	BFA	0.33	2,544,337	5.17	39,506,942	6.4	78.16	GENUS	87
Peru	PER	0.19	2,340,912	6.91	83,177,886	2.8	69.15	GENUS	58
mibia	M	2.40	2,230,420	12.53	11,632,901	19.2	61.61	GENUS	
Guinea	GIN	0.43	2,075,939	2.87	13,750,757	15.1	45.66	GENUS	97
Austria	AUT	0.52	1,714,895	28.31	93,140,068	1.8	98.13	GENUS	10
Chi	CHN	0.00	1,711,143	15.79	8,300,536,782	0.0	90.27	GENUS	35
Switzerland	CHE	0.42	1,334,269	20.92	66,121,410	2.0	93.11	GENUS	4
United Kingdom	GBR	0.05	1,170,366	24.35	603,768,499	0.2	91.38	GENUS	17
France	FRA	0.05	1,129,570	25.52	608,339,578	0.2	112.11	GENUS	16
Portugal	PRT	0.29	1,081,960	25.11	93,531,745	1.2	100.64	GENUS	20
Denmark	DNK	0.46	971,146	22.19	46,947,588	2.1	131.09	GENUS	14
Spain	ESP	0.06	962,316	25.95	443,068,592	0.2	95.10	GENUS	25
Italy	ITA	0.04	896,112	24.91	550,031,244	0.2	106.46	GENUS	23
Romania	ROU	0.13	892,374	14.93	104,892,909	0.9	97.39	GENUS	38
Poland	POL	0.06	859,378	20.24	279,839,753	0.3	100.54	GENUS	24

Norway	NOR	0.41	815,329	18.96	37,546,579	2.2	99.49	GENUS	5
Netherlands	NLD	0.11	668,847	23.98	150,102,282	0.4	126.77	GENUS	9
Gambia	GMB	0.61	542,376	3.11	2,747,366	19.7	57.79	GENUS	
Belgium	BEL	0.10	430,253	21.50	91,008,519	0.5	102.10	GENUS	15
Mauritius	MUS	0.42	195,792	17.52	8,136,858	2.4	75.25	GENUS	
Slovakia	SVK	0.08	169,304	17.03	33,950,672	0.5	73.98	GENUS	47
Czech Republic	CZE	0.04	151,162	23.15	90,538,968	0.2	86.36	GENUS	32
Uruguay	URY	0.10	127,309	22.95	29,122,487	0.4	106.62	GENUS	33
Cyprus	CYP	0.28	124,848	22.96	10,126,283	1.2	75.13	GENUS	
Tunisia	TUN	0.03	117,411	8.37	36,133,443	0.3	94.18	GENUS	69
Finland	FIN	0.05	105,026	20.63	41,743,592	0.3	107.21	GENUS	5
Greece	GRC	0.02	65,717	23.19	88,267,742	0.1	115.18	GENUS	31
Luxembourg	LUX	0.24	54,537	27.58	6,305,412	0.9	110.09	GENUS	
Ireland	IRL	0.03	52,479	22.36	40,323,527	0.1	135.51	GENUS	2
Senegal	SEN	0.00	25,600	4.71	28,811,554	0.1	57.21	GENUS	81
Lithuania	LTU	0.02	22,689	20.05	19,935,347	0.1	131.22	GENUS	
Slovenia	SVN	0.02	14,360	22.42	17,020,490	0.1	89.64	GENUS	
Kazakhstan	KAZ	0.00	10,383	21.71	148,906,158	0.0	90.42	GENUS	48
UAE	ARE	0.00	8,964	16.89	61,030,837	0.0	98.34	GENUS	21
Bulgaria	BGR	0.00	5,302	16.31	41,393,014	0.0	78.60	GENUS	51
Malta	MLT	0.01	1,615	25.02	4,034,812	0.0	98.95	GENUS	
Russian Federation	RUS	0.00	677	19.62	1,045,774,988	0.0	92.85	GENUS	42
Ecuador	ECU	-	16,250,000	17.58	113,282,734	-	71.48	Halpern et al 20	63
Georgia	GEO	-	6,500	7.83	11,415,074	-	97.50	FAO	
Bahamas	BHS	-	2,080	28.55	4,100,189	-	63.39	FAO	
Indonesia	IDN	-	1,950	3.81	380,485,161	-	59.16	FAO	62
Cabo Verde	CPV	-	1,430	15.24	3,094,569	-	69.69	FAO	
Albania	ALB	-	260	12.88	13,542,886	-	94.09	FAO	
Guya	GUY		16,250,000	13.04	3,746,473	433.7		Halpern et al 2019	
Surime	SUR		16,250,000	17.58	3,767,034	431.4		Halpern et al 2019	
Bolivia	BOL		16,250,000	21.52	91,753,350	17.7		Halpern et al 20	75
Colombia	COL		16,250,000	15.19	282,250,624	5.8		Halpern et al 2019	
Venezuela	VEN		16,250,000	27.93	290,112,828	5.6		Halpern et al 20	113
Brazil	BRA		16,250,000	29.55	2,294,403,831	0.7		Halpern et al 20	39
Zambia	ZMB		4,940,000	-	-			FAO	101
Gabon	GAB		3,315,390	-	-			FAO	
Afghanistan	AFG		1,040,000	-	-			FAO	
Liberia	LBR		1,040,000	-	-			FAO	
Togo	TGO		733,980	-	-			FAO	102
Lesotho	LSO		676,000	-	-			FAO	
Chad	TCD		585,000	-	-			FAO	109
Sierra Leone	SLE		396,760	-	-			FAO	106

Table S1. Estimated annual wild meat consumption and food security indices for 83 countries with non-zero estimates. Related to Figure 1.

Country	ISO3	Estimated extra pasture (km2)	Estimated extra crop land (km2)	Total estimated extra agricultural land (km2)	Estimated number of species destined for extinction
Ecuador	ECU	6082.2	1262.7	7344.9	85.1
Colombia	COL	6711.1	1284.7	7995.8	41.8
United States	USA	8473.3	3808.4	12281.7	24.8
Venezuela	VEN	4274	1110	5384.1	15.1
Cote d'Ivoire	CIV	5302.8	1532.4	6835.2	12.4
Cameroon	CMR	3104	754.2	3858.2	10
Brazil	BRA	7857.2	1380.8	9238	8.2
Nigeria	NGA	8617.5	1702.1	10319.6	6.3
Suriname	SUR	3853.2	1048.7	4901.9	6.2
Bolivia	BOL	5759.8	1188	6947.7	5.9
Guyana	GUY	1497.4	803.1	2300.5	5.3
Madagascar	MDG	322.8	87.6	410.5	4.8
Ghana	GHA	1556.1	657.7	2213.7	3.7
Rwanda	RWA	863.5	177.8	1041.3	3.6
Ethiopia	ETH	6351.7	1033	7384.7	3.2
Morocco	MAR	1449.4	425.1	1874.5	3.2
Argentina	ARG	4106.7	627.3	4734	2.8
New Zealand	NZL	1198.5	217.6	1416.1	2.8
South Africa	ZAF	1142	447	1589	2.5
Tanzania	TZA	1244.8	248.8	1493.6	1.9
Germany	DEU	1087.6	754.3	1841.8	1.9
Gabon	GAB	355.4	237.9	593.3	1.6
Peru	PER	263.9	65.8	329.7	1.4
Liberia	LBR	73.4	77.2	150.5	1.4
Kenya	KEN	1968.1	338.7	2306.7	1.3
Congo - Brazzaville	COG	330.9	359.5	690.4	1.3
Mauritius	MUS	9.9	5.5	15.4	0.8
China	CHN	111.2	85.2	196.4	0.7
Portugal	PRT	84.1	52	136.1	0.7
Iran	IRN	161.4	66.8	228.1	0.5
Italy	ITA	88.4	47.9	136.4	0.5
Spain	ESP	63	48	111	0.5
Zambia	ZMB	1796.5	455.4	2251.9	0.4
Austria	AUT	99.4	65.9	165.3	0.4
Sierra Leone	SLE	101.6	31.2	132.8	0.4
Zimbabwe	ZWE	1289.3	391.8	1681.1	0.4
Guinea	GIN	361.3	68.4	429.6	0.4
Switzerland	CHE	90	45	135	0.3
Central African Republic	CAF	1359	253.8	1612.8	0.3
Sweden	SWE	336.8	200.9	537.7	0.3
Lesotho	LSO	264.2	57.6	321.8	0.3
Cyprus	CYP	8.1	5.9	13.9	0.2
France	FRA	92.2	42.8	135.1	0.2
Botswana	BWA	1216.1	253.4	1469.6	0.2
Romania	ROU	68.9	47.6	116.5	0.2
Afghanistan	AFG	556.7	84.9	641.6	0.1
Togo	TGO	157.2	54.3	211.5	0.1
Namibia	NAM	250.1	66.7	316.8	0.1
Poland	POL	10.7	46	56.6	0.1
Angola	AGO	178.2	93.1	271.3	0.1
Denmark	DNK	86	40.9	126.9	0.1
Norway	NOR	176.2	77.7	253.9	0.1
United Kingdom	GBR	83.8	49.7	133.5	0.1
Mali	MLI	1725	257.7	1982.8	0.1
Benin	BEN	140.8	73	213.8	0
Niger	NER	2107.6	325	2432.7	0
Belgium	BEL	26.7	16	42.7	0
Netherlands	NLD	22.5	12.8	35.3	0
Greece	GRC	11.6	3.5	15.1	0
Burkina Faso	BFA	380.9	74.7	455.6	0
Sudan	SDN	803.8	101.9	905.7	0
Slovakia	SVK	4.5	8	12.5	0
Tunisia	TUN	18.4	4.4	22.8	0
Czechia	CZE	5	8	13	0
Gambia	GMB	39.3	12.5	51.8	0
Georgia	GEO	1.1	0.5	1.6	0

Luxembourg	LUX	8.3	4.3	12.7	0
Chad	TCD	361.1	56.3	417.4	0
Cape Verde	CPV	0.1	0.1	0.2	0
Indonesia	IDN	0.1	0.3	0.3	0
Finland	FIN	6.8	4.9	11.7	0
Slovenia	SVN	1.3	0.7	2.1	0
Bahamas	BHS	0.2	0.2	0.4	0
Uruguay	URY	12.1	2	14.2	0
Ireland	IRL	5.2	2.9	8.1	0
Lithuania	LTU	0.4	1.3	1.7	0
Bulgaria	BGR	0.2	0.3	0.5	0
Malta	MLT	0.1	0.1	0.2	0
Kazakhstan	KAZ	1.9	0.5	2.3	0
Senegal	SEN	3.3	0.7	4	0
Albania	ALB	0.1	0	0.1	0
United Arab Emirates	ARE	0.7	0.3	1	0
Russia	RUS	0.1	0	0.1	0

Table S2. Estimated land demand and biodiversity loss per country. Related to Figure 1.

Case study	Key characteristics	Key refs
Madagascar	**Current consumption**: High dependence on wild meat for nutrition and food security. Around Ankarafantsika National Park ~90% of households hunt wildlife at least once per week to cope with food insecurity.<br>**Environmental factors**: An island nation, with ~71% of land cultivated, and ~10% designated as protected. Other suitable forests and hillsides continue to be cleared, primarily for small-scale farming, but space for further expansion is limited. Conservation restrictions to stop forest clearance and hunting already result in significant welfare costs to communities.<br>**Socio-economic factors:** Food insecurity and malnutrition is high, poverty (as opposed to wealth) mostly drives wild meat consumption<br>**Overall assessment:** Food system would struggle to adapt to loss of wild meat - protein intake would likely be reduced for many rural households, which are already food insecure, leading to malnutrition. Alternatively, high social costs may lead to non-compliance with prohibitions, as is already the case in/around existing protected areas.	S1-4
East Region, Cameroon	**Current consumption**: Wild meat important for diets and nutrition, particularly in rural areas: 30-80% of protein intake in rural households<br>**Environmental factors**: Rural agriculture is subsistence and seasonal. Examples of viable alternatives to wild meat hunting in rural areas remain elusive. Small-scale aquaculture is under-developed, but requires major investment in capacity and capital, and may be unsuitable.<br>**Socio-economic factors**: Established cultural preferences for wild meat. Capacity to enforce regulations is weak in remote areas.<br>**Overall assessment**: Rural food system would struggle to adapt to loss of wild meat from diets, due to lack of space and resources for alternatives. Prohibitions may be socially illegitimate and difficult to enforce.	S5-7
Malawi	**Current consumption:** On average of 14% of households hunt wildlife, 21% of households consume wild meat. Hunting and consumption of wildlife is already illegal in many areas, but continues via illicit and informal markets.<br>**Environmental factors**: Agricultural production is seasonal, space for agricultural expansion is severely limited.<br>**Socio-economic factors**: Hunting is a cultural tradition in the Northern region, and there are taste preferences for wild meat over domestic meat. Households hunt for income and subsistence. Malawi is food insecure, and wild meat makes up gaps in food availability during the agricultural lean season. Supply chains for alternative protein sources are weak.<br>**Overall assessment**: Rural food system would struggle to adapt to loss of wild meat, and any additional prohibitions are likely to be socially illegitimate, with persistence of informal markets. Though urban Malawians consuming wild meat (mice and birds) as a delicacy may adapt.	S8-10
Rural Gabon	**Current consumption:** >70% of rural families participate in subsistence hunting, 40-60% of households sell wild meat, wild meat provides up to 90% of dietary protein in some families. Some evidence of declining hunting due to urbanisation, though there are peaks during seasonal employment gaps, and rural people remain highly dependent on forest products.<br>**Environmental factors**: Agricultural production is small-scale and seasonal<br>**Socio-economic factors**: Wild meat is a deeply-rooted cultural preference with inelastic demand. Relatively wealthier households (even in poor rural areas) consume more, and people are willing to pay more for wild meat than livestock. Hunting can make up one quarter of household income in some areas, and remote villages have low capacity to change livelihood strategies, with ability to adapt depending on proximity to facilities and infrastructure, and availability of resources.<br>**Overall assessment**: Rural food system would struggle to adapt to loss of wild meat from diets and livelihoods. Though urbanisation may reduce participation in hunting, demand for wild meat may remain or increase due to increased wealth and cultural preferences. Prohibitions may be socially illegitimate and difficult to enforce, even with livestock alternatives.	S11-15
Brazilian Amazon	**Current consumption:** Subsistence hunting (and fishing) is an important cultural activity and major source of dietary protein for indigenous and rural communities in remote areas of Amazonia. Estimated that 89,000 tons of wild meat are consumed per year by 8 million peoples in Brazilian Amazonia. Wild meat provides 8-72% of total protein consumed by Amazonian people, depending on socio-ecological systems. In urban centres of the interior of Amazonia, > 80% of households consume wild meat.<br>**Environmental factors:** Well-established large-scale agriculture has led to high rates of deforestation. 44% of remaining natural habitat is protected, in to which large-scale agriculture cannot expand (though could feasibly expand only ~20% of non-protected forest remnants).<br>**Socio-economic factors:** Well-established large-scale agriculture and cattle ranching in the Amazonian deforestation frontier. Market is aimed at national and international consumers, and does not supply remote rural and indigenous communities. Although hunting is permitted for indigenous peoples, uncertain legal status of hunting leaves other rural populations subject to arbitrary interpretation and weak enforcement of contradictory laws, contributing to informality and illicit markets. In urban centers prices of wild meat, chicken, and beef vary according to availability and distance to productions areas. Limited evidence that	S16-19

	incentives and social marketing can encourage alternatives (chicken) in urban centres. However, livestock raising for food provision is not common amongst rural and indigenous communities, with many previous husbandry initiatives failing for logistical, technical, social and environmental reasons.<br>**Overall assessment**: Rural and indigenous food system would be unable to rapidly adapt to loss of wild meat, primarily due to poor access to alternatives, but also due to cultural importance of wild meat. Communities would likely rely even more on fishing, since it is the most complementary protein source in most of Amazonia. Agricultural expansion may occur to increase protein supply to urban consumers. The social costs in terms of lost rights and traditions would be high, and prohibitions would be difficult to enforce. Community-based sustainable hunting of certain low-disease-risk species may represent a more viable and socially-just option.	
**Brazilian Atlantic Forest**	**Current consumption:** Mostly rural communities who hunt wildlife for diet complementation (not strictly subsistence), recreation, retaliation and trade to urban areas. In Southern Bahian ~50% of rural households hunt occasionally in protected areas primarily for consumption. Subsistence hunting is an important cultural activity and a source of animal protein for ~ 167000 indigenous. Sport and commercial hunting are also performed by urban residents. Hunting is already illegal except for satisfying hunger of a person and for indigenous peoples in officially recognized territories, though enforcement is limited, so illegal hunting continues including in strictly protected areas.<br>**Environmental factors:** Well-established agricultural sector (which could potentially intensify production) and urbanisation. A biodiversity hotspot where ~28% of original vegetation cover remains (highly dispersed and fragmented), and is under continued pressure from hunting, logging and agricultural expansion. Only ~30% of remaining forest is protected.<br>**Socio-economic factors:** Small-scale agriculture and animal husbandry are common in rural areas. Intense urbanization and access to markets mean most people can access alternative protein sources. However, cultural aspects and taste preference for wild meat are high in some areas.<br>**Overall assessment**: The food system could potentially adapt to removal of wild meat. However investments would be necessary to sustainably intensify current production and/or recover degraded areas to expand agriculture, so avoiding further deforestation and threats to biodiversity in this already highly fragmented region. The social costs would be high for the rural poor and for indigenous populations also in terms of lost rights and traditions. Existing prohibitions are already difficult to enforce, additional regulations (affecting the most vulnerable populations) may be socially unjust and result in non-compliance.	S20-22
**Tropical south west Ghana**	**Current consumption:** In rural areas ~44% of households consume wild meat on a weekly basis and ~40% engage in hunting (though not as a key livelihood). In urban areas, ~69% of people report eating wild meat, though few (6%) on a daily basis. The importance of wild meat for consumers and hunters appears to be declining, though remains an important commodity for some, particularly the rural poor.<br>**Environmental factors:** Scope for agricultural expansion into primary forests may be limited due to already highly fragmented habitat. Any increased livestock production will intensify competition in the existing agricultural landscape, with potential for escalating conflict between herders and farmers with severe social and economic consequences.<br>**Socio-economic factors:** Consumer surveys suggest preferences for wild meat are declining in urban areas, though remains an important, high-value cultural commodity. Hunting and trade is an important economic activity - it serves a safety net function during seasonal periods of economic hardship, and those involved are often vulnerable groups and indicate they are unable/unwilling to change.<br>**Overall assessment:** Ghana's food system could potentially adapt to loss of wild meat overall; however severe impacts would be felt by some sectors of society. In rural settings, both consumption and reliance on wild meat for income is greatest, and these communities are the least able to adapt to shocks. An economic shock may be the biggest risk, especially in light of the well-developed commercial trade in wild meat. Female traders and wholesalers who often derive their entire income and livelihood from wild meat are likely to be most affected.	S15-S23-26
**USA**	**Current consumption**: Large absolute volumes of wildmeat consumption. 13.7 million Americans participate in hunting, with food-motivated hunting particularly high in rural areas, driven by preferences for wild meat and limited access to/high prices for commercial meat<br>**Environmental factors**: Agricultural systems are high-yielding and adaptive - could expand or adapt in some areas, though may lead to biodiversity losses in-country, or displacement effects on other countries if cheaper products are imported<br>**Socio-economic factors**: On average, Americans are not lacking protein, however reliance on wild meat and availability of alternatives is heterogenous. Wild meat consumption is higher in rural areas and socially-marginalised communities.<br>**Overall assessment**: Removal of wild meat would mainly impact rural and relatively food-insecure groups. Agricultural expansion may occur, and would need to target rural areas, with a focus on improved supply chains. The hunting industry – and revenues generated for conservation – would suffer large economic losses. Recreational hunters and those with taste preferences and strong attachments to hunting would suffer social costs, and may not comply with prohibitions. Continued sustainable hunting would likely be more beneficial overall.	S27
**China**	**Current consumption:** Estimated that ~12% of total population consume wildlife, though as high as 60% in some regions (e.g. SW China). However, a recent survey shows that >90% of people are against consumption and trade of wildlife following COVID-19.	S28-30

	**Environmental factors:** Agriculture is high-yielding in some areas, though large population and rapidly growing demand means domestic livestock production cannot meet current demand - imports of livestock and feed are increasing, with displacement effects on other countries<br>**Socio-economic factors**: Wild meat is typically consumed for taste, rarity, nourishment and social purposes. Over 14 million people directly employed in wildlife farming industry, which forms a key part of the rural economy, and was once encouraged by the government as part of the poverty alleviation measures. Of these, around 6.3 million people are employed in wildlife farming for human consumption. However, >90% of educated urban people support more stringent regulation of wildlife consumption and trade following COVID-19.<br>**Overall assessment:** China's food system could potentially adapt to loss of wild meat overall, though increases in agricultural production (or imports from elsewhere) will be required, with risks to biodiversity and EIDs. However, given the role of wildlife farming in providing employment for rural people, there could be significant economic shocks in rural areas. If farms are closed without rapid investment in new economic activities or shifting of eating habits, people may turn to illegal hunting and trading of wild species, and/or agriculture, with implications for biodiversity loss. Continued faming of low-risk species (e.g. reptiles, amphibians) would likely be more beneficial overall.	
**Nigeria**	**Current consumption:** Communities in close proximity to wildlife regularly hunt, process and/or consume wild meat (e.g. >99% of people hunt and consume around Cross River National Park, >52% of people hunt and sell around Old Oyo National Park, 11% hunt in Otukpo)<br>**Environmental factors:** Well-developed, high-value agriculture sector in Nigeria, which could be expanded or intensified. Extremely high rates of tropical deforestation, driven to a large degree by agricultural expansion, and continued pressure on remaining tropical forest, which is both a hotspot of mammal biodiversity and EID risk. Seasonality strongly influences hunting – preferable conditions in dry season.<br>**Socio-economic factors**: Wild meat is used for food, income, taste and cultural reasons. Studies show preferences for wild meat over domestic meat. However, hunting is considered an undesirable livelihood – it is challenging, people are aware of zoonotic disease risks, and indicate willingness to change with provision of alternatives. Evidence that Ebola-related campaign discouraged wild meat consumption.<br>**Overall assessment:** Nigeria's food system could potentially adapt to loss of wild meat through expansion of animal agriculture and provision of alternatives to rural communities, though with concomitant risks for biodiversity and EIDs. Taste preferences for wild meat over domestic meat would remain a challenge, though public health messaging may overcome this. Alternative protein sources that satisfy taste preferences – such as small-scale wildlife farming or sustainably managed hunting of low-disease-risk species – may be more effective.	S31-33

Table S3. Detailed information on case study places. Related to Table 1.

Supplemental references

S1. Merson, S.D., Dollar, L.J., Johnson, P.J., and Macdonald, D.W. (2019). Poverty not taste drives the consumption of protected species in Madagascar. Biodivers Conserv *28*, 3669–3689.

S2. Borgerson, C., Randrianasolo, J.F., Andraina, T.R., Anjaranirina, E.J.G., Randriamady, H.J., Merson, S., Dollar, L., and Golden, C.D. (2020). Wildlife hunting in complex human-environmental systems: How understanding natural resource use and human welfare can improve conservation in the Ankarafantsika National Park, Madagascar. Madagascar Conserv Dev *14*, 37–45.

S3. Poudyal, M., Jones, J.P.G., Rakotonarivo, O.S., Hockley, N., Gibbons, J.M., Mandimbiniaina, R., Rasoamanana, A., Andrianantenaina, N.S., and Ramamonjisoa, B.S. (2018). Who bears the cost of forest conservation? PeerJ *2018*, e5106.

S4. Jenkins, R.K.B., Keane, A., Rakotoarivelo, A.R., Rakotomboavonjy, V., Randrianandrianina, F.H., Razafimanahaka, H.J., Ralaiarimalala, S.R., and Jones, J.P.G. (2011). Analysis of patterns of bushmeat consumption reveals extensive exploitation of protected species in eastern madagascar. PLoS One *6*, 1–11.

S5. Koppert, G.J., Dounias, E., Froment, A., and Pasquet, P. (1996). Food consumption in three forest populations of the southern coastal area of Cameroon : Yassa - Mvae - Bakola. In Tropical Forests, People and Food: Biocultural Interactions And Applications to Development, C. Hladik, O. Linares, H. Pagezy, A. Semple, and M. Hadley, eds. (UNESCO), pp. 295–310.

S6. Booker, F. (2019). Why Eat Wild Meat? Results of a literature review on drivers of wild meat as a food choice.

S7. Kleinschroth, F., Laporte, N., Laurance, W.F., Goetz, S.J., and Ghazoul, J. (2019). Road expansion and persistence in forests of the Congo Basin. Nat Sustain *2*, 628–634.

S8. van Velden, J.L., Travers, H., Moyo, B.H.Z., and Biggs, D. (2020). Using scenarios to understand community-based interventions for bushmeat hunting and consumption in African savannas. Biol Conserv *248*, 108676.

S9. Maseko, H., Shackleton, C.M., Nagoli, J., and Pullanikkatil, D. (2017). Children and Wild Foods in the Context of Deforestation in Rural Malawi. Hum Ecol *45*, 795–807.

S10. van Velden, J.L., Wilson, K., Lindsey, P.A., McCallum, H., Moyo, B.H.Z., and Biggs, D. (2020). Bushmeat hunting and consumption is a pervasive issue in African savannahs: insights from four protected areas in Malawi. Biodivers Conserv *29*, 1443–1464.

S11. Wilkie, D.S., Starkey, M., Abernethy, K., Effa, E.N., Telfer, P., and Godoy, R. (2005). Role of prices and wealth in consumer demand for bushmeat in Gabon, Central Africa. Conserv Biol *19*, 268–274.

S12. van Vliet, N., and Nasi, R. (2008). Hunting for livelihood in Northeast Gabon: Patterns, evolution, sustainability. Ecol Soc *13*.

S13. Abernethy, K., and Ndong Obiang, A.M. (2010). Bushmeat in Gabon.

S14. Mcnamara, J., Robinson, E.J.Z., Abernethy, K., Midoko, D., Hannah, I., and Juliet, N.K.S. (2020). COVID - 19 , Systemic Crisis , and Possible Implications for the Wild Meat Trade in Sub - Saharan Africa. Environ Resour Econ.

S15. Van Gils, E.J.T., Ingram, V.J., Iponga, D.M., and Abernethy, K. (2019). Changes in Livelihood Practices, Strategies and Dependence on Bushmeat in Two Provinces in Gabon. Int For Rev *21*, 108–127.

S16. Chaves, W.A., Valle, D.R., Monroe, M.C., Wilkie, D.S., Sieving, K.E., and Sadowsky, B. (2018). Changing Wild Meat Consumption: An Experiment in the Central Amazon, Brazil. Conserv Lett *11*, 1–10.

S17. Antunes, A.P., Rebêlo, G.H., Pezzuti, J.C.B., Vieira, M.A.R. de M., Constantino, P. de A.L., Campos-Silva, J.V., Fonseca, R., Durigan, C.C., Ramos, R.M., Amaral, J.V. do, et al. (2019). A conspiracy of silence: Subsistence hunting rights in the Brazilian Amazon. Land use policy *84*, 1–11.

S18. Peres, C.A. (2000). Effects of Subsistence Hunting on Vertebrate Community Structure in Amazonian Forests.

S19. El Bizri, H.R., Morcatty, T.Q., Valsecchi, J., Mayor, P., Ribeiro, J.E.S., Vasconcelos Neto, C.F.A., Oliveira, J.S., Furtado, K.M., Ferreira, U.C., Miranda, C.F.S., et al. (2020). Urban wild meat consumption and trade in central Amazonia. Conserv Biol *34*, 438–448.

S20. Strassburg, B.B.N., Beyer, H.L., Crouzeilles, R., Iribarrem, A., Barros, F., de Siqueira, M.F., Sánchez-Tapia, A., Balmford, A., Sansevero, J.B.B., Brancalion, P.H.S., et al. (2019). Strategic approaches to restoring ecosystems can triple conservation gains and halve costs. Nat Ecol Evol *3*, 62–70.

S21. Castilho, L.C., De Vleeschouwer, K.M., Milner-Gulland, E.J., and Schiavetti, A. (2019). Hunting of

mammal species in protected areas of the southern Bahian Atlantic Forest, Brazil. ORYX *53*, 687–697.
S22. Sousa, J.A.C., and Srbek-Araujo, A.C. (2017). Are we headed towards the defaunation of the last large Atlantic Forest remnants? Poaching activities in one of the largest remnants of the Tabuleiro forests in southeastern Brazil. Environ Monit Assess *189*, 1–13.
S23. Brashares, J.S., Golden, C.D., Weinbaum, K.Z., Barrett, C.B., and Okello, G. V. (2011). Economic and geographic drivers of wildlife consumption in rural Africa. Proc Natl Acad Sci U S A *108*, 13931–13936.
S24. Shanti-Alexander, J., McNamara, J., Rowcliffe, J.M., Oppong, J., and Milner-Gulland, E.J. (2015). The role of bushmeat in a West African agricultural landscape. ORYX *49*, 643–651.
S25. Schulte-Herbrüggen, B., Cowlishaw, G., Homewood, K., and Rowcliffe, J.M. (2013). The Importance of Bushmeat in the Livelihoods of West African Cash-Crop Farmers Living in a Faunally-Depleted Landscape. PLoS One *8*, e72807.
S26. McNamara, J., Rowcliffe, M., Cowlishaw, G., Alexander, J.S., Ntiamoa-Baidu, Y., Brenya, A., and Milner-Gulland, E.J. (2016). Characterising wildlife trade market supply-demand dynamics. PLoS One *11*.
S27. Conservation Visions Inc (2016). Consumption patterns of wild protein in North America: a literature review in support of the wild harvest initiative.
S28. Wang, H., Shao, J., Luo, X., Chuai, Z., Xu, S., Geng, M., and Gao, Z. (2020). Wildlife consumption ban is insufficient. Science (80-) *367*, 1435–1435.
S29. Zhang, L., Hua, N., and Sun, S. (2008). Wildlife trade, consumption and conservation awareness in southwest China. Biodivers Conserv *17*, 1493–1516.
S30. Shi, X., Zhang, X., Xiao, L., Li, B. V, Liu, J., Yang, F., Zhao, X., and Cheng, C. (2020). Public perception of wildlife consumption and trade during the COVID-19 outbreak. Biodivers Sci *28*, 630–643.
S31. Halidu, S.K. (2019). Assessment of bush meat sale and its implication on wildlife conservation in Old Oyo National Park , Nigeria. World News Nat Sci *23*, 266–275.
S32. Friant, S., Paige, S.B., and Goldberg, T.L. (2015). Drivers of Bushmeat Hunting and Perceptions of Zoonoses in Nigerian Hunting Communities. PLoS Negl Trop Dis *9*, 1–16.
S33. Fa, J.E., Seymour, S., Dupain, J., Amin, R., Albrechtsen, L., and Macdonald, D. (2006). Getting to grips with the magnitude of exploitation: Bushmeat in the Cross-Sanaga rivers region, Nigeria and Cameroon. Biol Conserv *129*, 497–510.
